# Comparative Sequence and Structural Analyses of G-Protein-Coupled Receptor Crystal Structures and Implications for Molecular Models

**DOI:** 10.1371/journal.pone.0007011

**Published:** 2009-09-16

**Authors:** Catherine L. Worth, Gunnar Kleinau, Gerd Krause

**Affiliations:** Leibniz-Institut für Molekulare Pharmakologie (FMP), Berlin, Germany; New Mexico State University, United States of America

## Abstract

**Background:**

Up until recently the only available experimental (high resolution) structure of a G-protein-coupled receptor (GPCR) was that of bovine rhodopsin. In the past few years the determination of GPCR structures has accelerated with three new receptors, as well as squid rhodopsin, being successfully crystallized. All share a common molecular architecture of seven transmembrane helices and can therefore serve as templates for building molecular models of homologous GPCRs. However, despite the common general architecture of these structures key differences do exist between them. The choice of which experimental GPCR structure(s) to use for building a comparative model of a particular GPCR is unclear and without detailed structural and sequence analyses, could be arbitrary. The aim of this study is therefore to perform a systematic and detailed analysis of sequence-structure relationships of known GPCR structures.

**Methodology:**

We analyzed in detail conserved and unique sequence motifs and structural features in experimentally-determined GPCR structures. Deeper insight into specific and important structural features of GPCRs as well as valuable information for template selection has been gained. Using key features a workflow has been formulated for identifying the most appropriate template(s) for building homology models of GPCRs of unknown structure. This workflow was applied to a set of 14 human family A GPCRs suggesting for each the most appropriate template(s) for building a comparative molecular model.

**Conclusions:**

The available crystal structures represent only a subset of all possible structural variation in family A GPCRs. Some GPCRs have structural features that are distributed over different crystal structures or which are not present in the templates suggesting that homology models should be built using multiple templates. This study provides a systematic analysis of GPCR crystal structures and a consistent method for identifying suitable templates for GPCR homology modelling that will help to produce more reliable three-dimensional models.

## Introduction

G-protein-coupled receptors (GPCRs) are the largest family of integral membrane receptors, transducing a wide variety of signals, and make up roughly 3% of genes in the human genome [1]. A vast number of mutations have been identified in GPCRs (both activating and inactivating) which are responsible for more than 30 different human diseases [2] such as cancer [3], [4], diabetes [5], hyperthyroidism [6], ovarian hyperstimulation syndrome [7], [8], congenital stationary night blindness [9] as well as being implicated in causing obesity [10]. It is estimated that 30–50% of current drug targets are GPCRs [11], [12], which is in contrast to the small proportion of genes in the human genome that are predicted to encode GPCRs, illustrating the importance of these proteins both medically and pharmaceutically.

Knowledge of the three-dimensional structure of GPCRs is important for understanding the molecular mechanism underlying diseases and syndromes caused by mutations in these receptors, as well as for the structure-based design of small molecules acting as therapeutic treatments. Currently structural data are restricted to four members of GPCR family A: Rhodopsin [13]–[17], Beta-1 adrenergic receptor [18], Beta-2 adrenergic receptor [19], [20] and Adenosine A2a receptor [21]. All were crystallised with inverse agonists or antagonists and therefore represent inactive conformations. The recent publication of the opsin structure [22] and opsin bound to a G-protein derived synthetic peptide [23] represent activated states, providing important information about the structural changes associated with activation of GPCRs. All of these GPCR structures are characterised by seven transmembrane helices (TMHs) and an eighth helix which lies approximately parallel to the intracellular membrane. Despite this conservation in overall architecture, the orientation and length of the helices vary to some extent [14], [17] and considerable structural diversity is observed in the three intracellular and extracellular loops [24], [25] that connect the seven TMHs [26]. Furthermore, differences are also observed in the orientation of sidechains (including highly conserved amino acids) [18], [27] and the presence and extent of helical distortions (kinks and bulges) [28].

Even with the recent progress that has been made in GPCR structural biology and reported improvements in GPCR expression protocols [29], [30], it is unlikely that the large gap in experimental GPCR structural space will be filled in the near future. To some extent however, the deficit in GPCR experimental structure data can be met by building molecular models of GPCRs of unknown structure by comparative (or homology) modelling. Up until 2007, comparative models of GPCRs had to be built using bovine rhodopsin as a template [31], [32]. Today there is the choice of five different GPCRs for building comparative models of GPCRs [33] in the inactive state and the two opsin structures for building comparative models of GPCRs in an active state.

Focusing on the aim of building a comparative model of a GPCR, it is not clear which GPCR structure(s) should be used as the template in order to maximise the accuracy of the model. This is an important issue as homology models have application in virtual screening studies, docking experiments (small molecule and protein-protein interactions) as well as being used to generate hypotheses about intra- and inter-molecular mechanisms. Hanson and Stevens have recently reviewed experimentally determined GPCR structures [26]. However, their structural analyses were brief and the implications for comparative model building were not addressed. The aim of this study is to provide a rational workflow for selecting the most appropriate template(s) for building comparative models of GPCRs with no experimentally determined structure. Here we have compared the available GPCR structures (in inactive conformations) and identified key distinguishing structural features. Combining these structural analyses with quantitative analyses of sequence similarities between the template structures has allowed us to develop workflows for template selection for each of the seven TMHs and helix 8. We have applied these workflows to an exemplary set of 14 GPCRs that are members of GPCR family A and which have been functionally characterised e.g. through mutagenesis experiments. Our results suggest that comparative models of GPCRs might be best built using a multiple template approach, producing chimeric GPCR models. This work provides the first rational analysis of available GPCR structures for homology modelling in light of the recent increase in available templates. Furthermore, our work provides a valuable protocol for producing more accurate and consistent GPCR models.

## Results

A set of potential template structures was created using five GPCRs with experimental structures (Table 1). A second set of GPCRs was created comprising 14 disease-associated proteins of unknown structure and for which mutation data are available (Table 2). These 14 GPCRs span the four main phylogenetic groups of GPCR family A: α (amine, opsin and MECA), β (peptides), γ (chemokine) and δ (glycoprotein and nucleotide receptors) [34].

**Table 1:** GPCRs with experimentally determined structures that were used in the analysis.

Protein name	Gene name	Species from	PDB code	Unique identifier
Adenosine-2A receptor	AA2AR	*Homo sapiens*	3EML	hAA2AR
Beta-1 adrenergic receptor	B1AR	*Meleagris gallopavo*	2VT4	tB1AR
Beta-2 adrenergic receptor	B2AR	*Homo sapiens*	2RH1	hB2AR
Rhodopsin	RHO	*Todarodes pacificus*	2Z73	sRHO
Rhodopsin	RHO	*Bos taurus*	1U19	bRHO

**Table 2:** GPCRs of unknown structure that were used in the analysis.

Protein name	Gene name	Species from	Unique identifier	Disease association
Rhodopsin	RHO	*Homo sapiens*	hRHO	Congenital night blindness [77], Retinitis pigmentosa [78]
Muscarinic acetylcholine receptor M1	ACM1	*Homo sapiens*	hACM1	Schizophrenia [79]
Dopamine D2 receptor	DRD2	*Homo sapiens*	hDRD2	Schizophrenia [80]
Vasopressin V1a receptor	V1AR	*Homo sapiens*	hV1AR	Autism [81]
Vasopressin V2 receptor	V2R	*Homo sapiens*	hV2R	Nephrogenic diabetes insipidus [5],
C-C chemokine receptor type 5	CCR5	*Homo sapiens*	hCCR5	Insulin-dependent diabetes mellitus type 22 [82]
Melanocortin receptor 4	MC4R	*Homo sapiens*	hMC4R	Obesity [10]
Cannabinoid receptor 1	CNR1	*Homo sapiens*	hCNR1	Obesity in men [83]
Cannabinoid receptor 2	CNR2	*Homo sapiens*	hCNR2	Osteoporosis [84]
P2Y purinoreceptor 1	P2RY1	*Homo sapiens*	hP2RY1	Thrombosis risk [85]
P2Y purinoreceptor 12	P2RY12	*Homo sapiens*	hP2RY12	Bleeding disorder [86]
Follicle-stimulating hormone receptor	FSHR	*Homo sapiens*	hFSHR	Ovarian hyperstimulation syndrome [7]
Lutropin-choriogonadotropic hormone receptor	LHCGR	*Homo sapiens*	hLHCGR	Leydig-cell tumour [3]
Thyroid-stimulating hormone receptor	TSHR	*Homo sapiens*	hTSHR	Hyperthyroidism [6], thyroid carcinoma [87]

### Structural diversity of the five known GPCR structures

Superimposing the five template GPCR structures using the seven highly conserved residues in the transmembrane helices as reference points resulted in root mean squared deviations (RMSDs) ranging from 0.63 Å (between Beta-1 adrenergic receptor [tB1AR] and Beta-2 adrenergic receptor [hB2AR]) to 4.03 Å (between bovine rhodopsin [bRHO] and squid rhodopsin [sRHO]) for the common core of the seven TMHs and helix 8 (Table S1 supporting information). This established method of superimposing GPCR structures [35] allowed us to quickly generate superimposed co-ordinates of the template structures that could then be used to improve a multiple sequence alignment (MSA).

**Figure 1:**
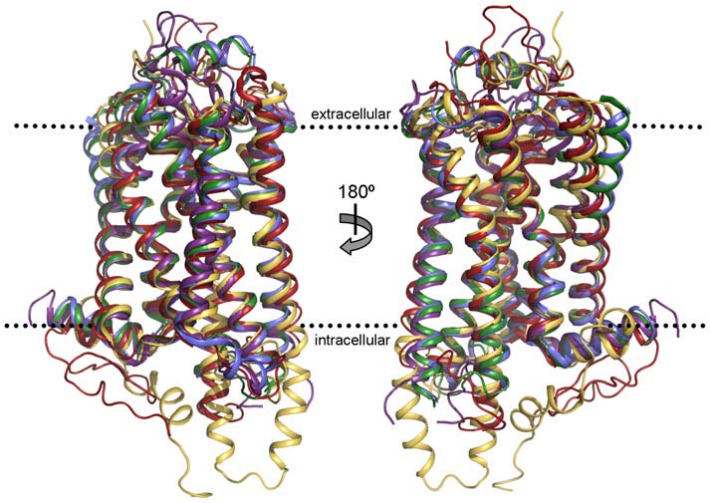
Structural superimposition of the five GPCR structures used in the analysis. The overall topology of the templates are remarkably similar, with the transmembrane helices superimposing relatively well in most cases (although there appears to be more variation at the extracellular side of membrane surface). hAA2AR is represented in purple, tB1AR in blue, hB2AR in green, sRho in yellow and bRho in red. All structure images were produced using Pymol [75].

**Figure 2:**
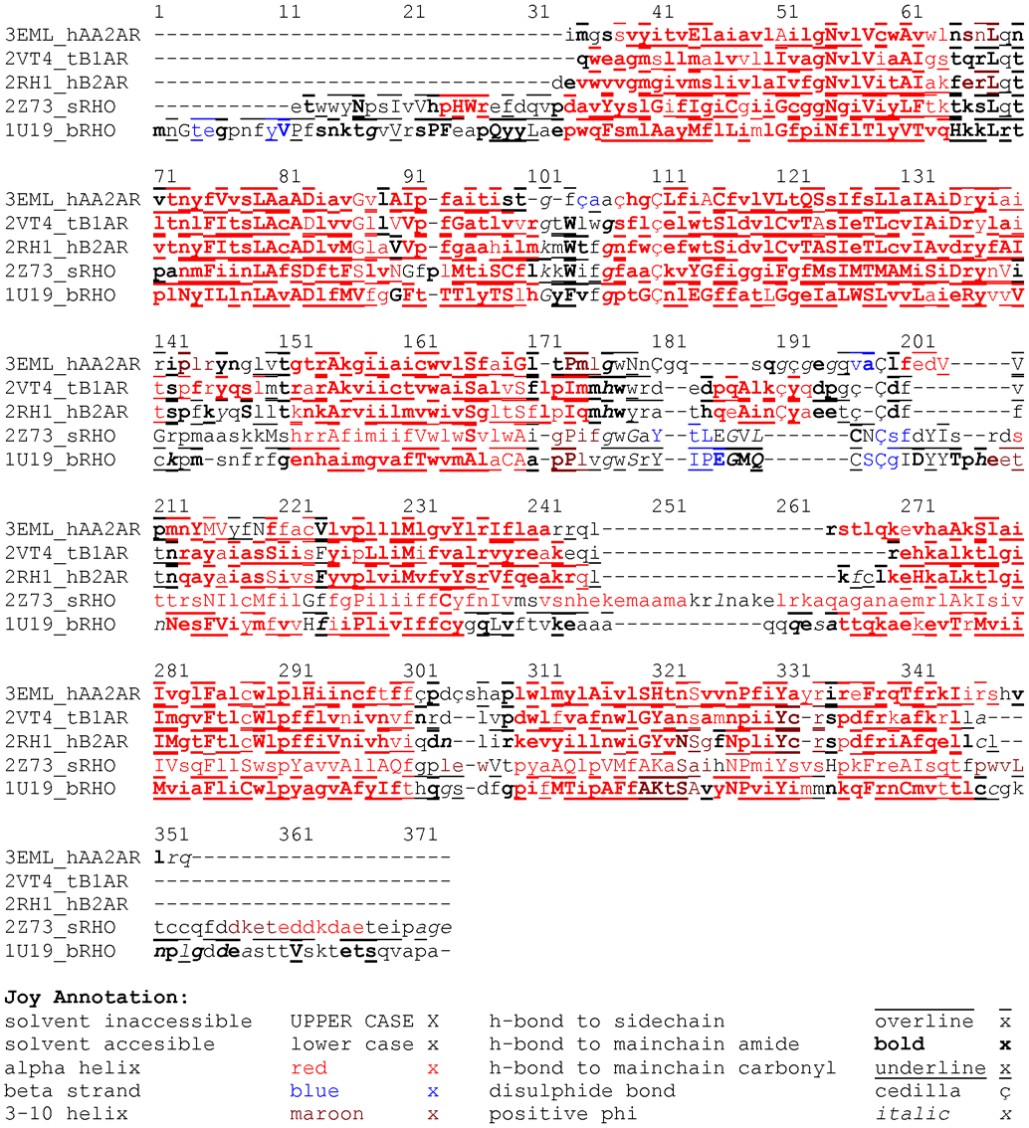
The structure-based sequence alignment of the five template GPCRs. The sequences correspond to the structures in the PDB files. The local structural environment of each residue (derived from the crystal structures) is displayed using JOY annotation [76]. The helical regions (shown in red) tend to be less variable than the loop regions (shown in black).

The identified boundaries of the seven TMHs and helix 8 (see Table S2) were used together with the superimposed structures to identify the common helical regions. It should be noted that carrying out superimposition of the five templates using the common helical regions improved the RMSDs obtained, with values ranging from 0.61 Å (between tB1AR and hB2AR) and 3.57 Å (between bRHO and sRHO) (Table S3). The transmembrane helices are relatively well conserved in conformation, with the intracellular and extracellular loops being much more variable (Figure 1)–this is also evident in the MSA of the five template structures (Figure 2) and the MSA of the five template structures and 14 target GPCRs (Figure S1, supporting information).

### Sequence similarity between template and target GPCRs

Apart from hRHO, the differences in sequence similarity between each target GPCR and the five template structures are small, ranging from 3% to 11% (Table **3**)–see materials and methods for a definition of percentage of sequence similarity. Like-wise, when restricting the comparisons to individual helices, the differences in sequence similarity between each target GPCR and the five templates structures are also small (Table 4 and supporting ), although the sequence similarity values of these helical regions tend to be higher.

**Table 3:** Sequence similarity scores between the 14 target GPCRs and five template structures for the region ranging from the start of TMH1 to the end of helix 8.

	hAA2AR	tB1AR	hB2AR	sRHO	bRHO
**hRHO**	38	37	36	49	**97**
**hACM1**	31	**34**	**34**	29	26
**hDRD2**	33	**38**	37	27	29
**hV1AR**	33	34	32	**36**	35
**hV2R**	31	34	33	**37**	35
**hCCR5**	36	**39**	**39**	36	38
**hMC4R**	41	**39**	**39**	34	32
**hCNR1**	**38**	37	35	34	31
**hCNR2**	37	**39**	35	35	32
**hP2RY1**	29	37	37	**40**	37
**hP2RY12**	31	**37**	35	31	32
**hFSHR**	**39**	32	35	37	35
**hLHCGR**	36	33	34	**37**	36
**hTSHR**	**37**	33	33	35	34

The highest scoring template(s) are indicated by bold font.

**Table 4:** Sequence similarity scores between each template structure and each target GPCRs for TMH2.

	hAA2AR	tB1AR	hB2AR	sRHO	bRHO
**hRHO**	46	40	40	36	**96**
**hACM1**	56	62	**65**	42	43
**hDRD2**	59	**62**	59	39	56
**hV1AR**	39	39	36	39	**45**
**hV2R**	**39**	36	33	**39**	**39**
**hCCR5**	**59**	**59**	56	42	43
**hMC4R**	46	43	**50**	45	56
**hCNR1**	42	45	**51**	48	36
**hCNR2**	36	45	39	**48**	30
**hP2RY1**	31	43	40	**45**	37
**hP2RY12**	**40**	**40**	37	33	37
**hFSHR**	43	50	**56**	39	34
**hLHCGR**	43	43	**50**	42	34
**hTSHR**	43	43	**46**	39	28

The highest scoring template(s) are indicated by bold font.

In conclusion, across the different TMHs and helix 8, there is no apparent consensus about which template has the highest sequence similarity (except for hRHO)–see Figure S2. These results indicate that there is no clear answer as to which template to use for homology model building based on sequence similarity alone. Therefore these results suggest that structural information needs to be included in the decision process of template selection for homology model building.

### Structural features to guide template selection

We performed detailed analyses of the superimposed three-dimensional structures of the five templates in order to identify structural features that could be incorporated into a modelling workflow. Features such as helix distortions (kinks and bulges), helix extensions, disulphide bridges and secondary structure within loops were considered. Comparison of these structural features in the five templates reveals three possibilities of occurrence (Table 5):

- Shared by all (such as Pro distortions in TMHs 4, 5, 6 and 7 and a conserved disulphide bridge between TMH3 and ECL2).
- Shared by a subset of the templates (such as a specific loop conformation).
- Some are unique to particular templates.

**Table 5:** Structural features observed in the five GPCR crystal structures.

		hAA2AR	tB1AR	hB2AR	sRho	bRho
TMH1	Pro distortion					+
	Gly-Gly bulge				+	
ICL1	3_10_ helix	+	+	+		
TMH2	Pro distortion	+	+	+	+	
	Gly-Gly distortion					+
	Bulge due to insertion				+	
ECL1	Disulphide bridge to ECL2	+				
	Beta-strand (indicated by above disulphide bridge)	+				
TMH3	Conserved Cys forms disulphide bridge to ECL2	+	+	+	+	+
	Second disulphide bridge to ECL2	+				
	Gly bend				+	+
ICL2	Helical	+	+			
	Tyr forming h-bond to Asp in DRY motif	+	+			
TMH4	Pro distortion	+	+	+	+	+
	Bulge due to insertion		+	+		
ECL2	Disulphide bridge to TMH3	+	+	+	+	+
	Beta-sheet				+	+
	Alpha-helix		+	+		
	Beta-strand	+				
	Intra-ECL2 disulphide bridge		+	+		
	Disulphide bridge to ECL1	+				
	Second disulphide bridge to TMH3	+				
TMH5	Pro kink	+	+	+	+	+
	Helix extension				+	
ICL3	Partial structure	+	+	+		
TMH6	Pro kink	+	+	+	+	+
	Helix extension				+	
ECL3	TMH6-ECL3 disulphide bridge	+				
	3_10_ helix				+	
TMH7	Pro kink	+	+	+	+	+
Helix 8	Insertion	+			+	+

+ indicates the presence of a particular structural feature.

**Figure 3:**
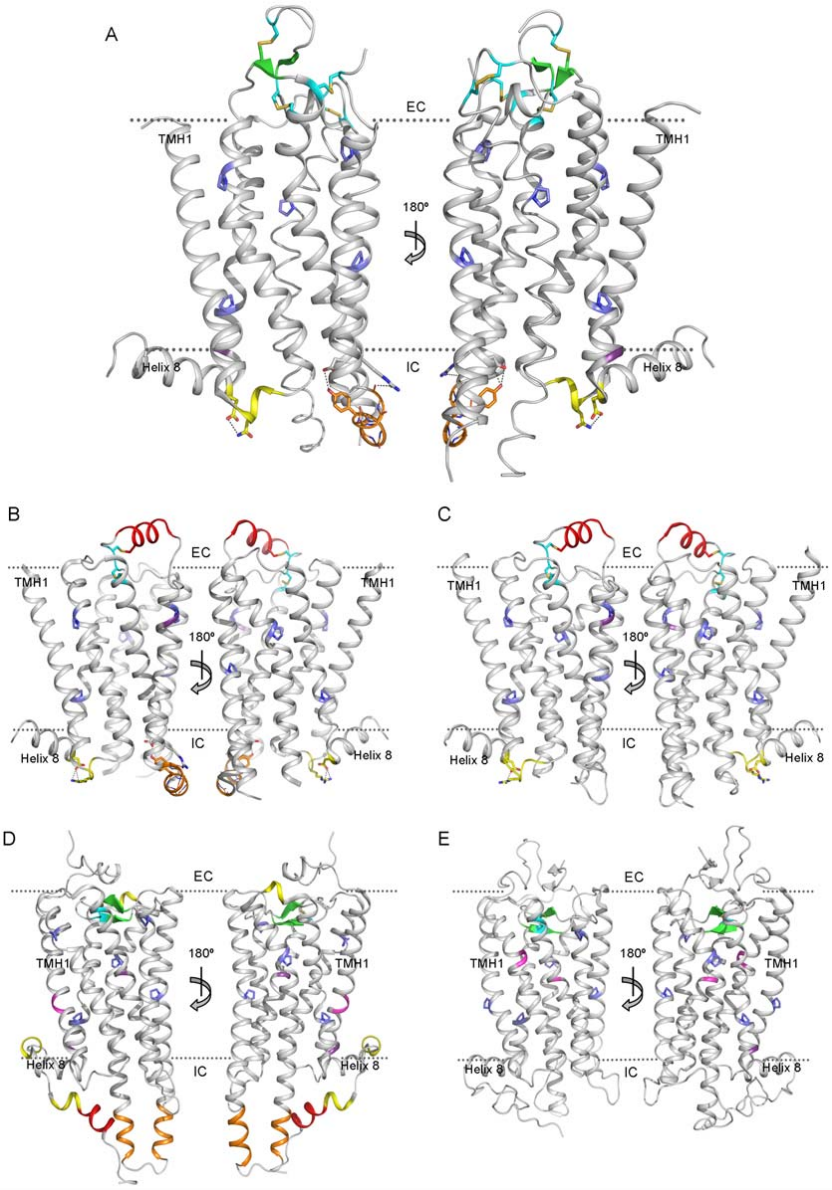
Key structural features identified in the five template GPCR structures. A) hAA2AR B) tB1AR C) hB2AR D) sRHO and E) bRHO. Features causing distortion of the transmembrane helices (TMHs) include: Pro distortions (sidechains shown in blue), insertions (backbone shown in purple) and Gly distortions (backbone shown in magenta). At the extracellular membrane side (EC) a number of disulphide bridges are observed (sidechains shown in turquoise) although only that formed by cysteine residues in TMH3 and ECL2 is conserved in all five templates. The β-strands formed by ECL1 and ECL2 in A (shown in green) are unique to this structure. ECL2 forms helical structures in B and C (shown in red) and β-sheets in D and E (shown in green). At the intracellular membrane side (IC), ICL1 (shown in yellow) and ICL2 (shown in orange) are helical in A–C and A–B respectively and are characterised by hydrogen bonds between polar sidechains. There are numerous helical structures that are unique to D: TMHs 5 and 6 are extended relative to the other structures (shown in orange); short 3_10_ helices are observed in ECL3 and after helix 8 (shown in yellow); an α-helix is observed at the C-terminal end of the polypeptide chain (shown in red). The membrane surfaces are indicated by a dashed line (approximate position).

**Figure 4:**
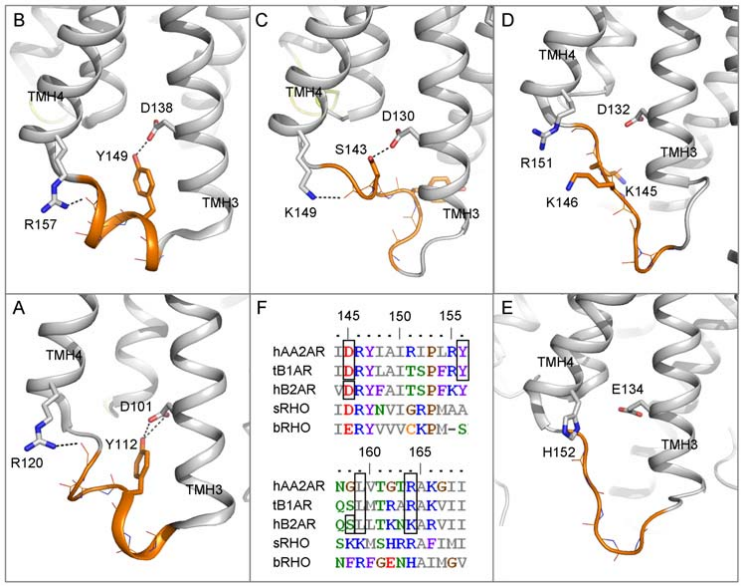
Structural and sequence diversity of ICL2 between the five template GPCRs. Both A) hAA2AR and B) tB1AR have helical structures (shown in orange) within ICL2. In both of these structures an Arg residue caps the ICL2 helix C-termini and a Tyr sidechain forms a hydrogen bond with an Asp sidechain in TMH3. This combination of constraining hydrogen bond interactions is not observed in C) hB2AR, D) sRHO and E) bRHO where ICL2 is of an irregular coil-like conformation (orange). F) Shows a section of the MSA covering ICL2 and the flanking helix termini. Those residues involved in hydrogen bond interactions in A–E are highlighted in grey boxes. It appears that the presence of both a Tyr at position 156 and an Arg at position 164 as well as the absence of a basic sidechain at position 159 can be used as markers for the presence of helical structures in ICL2.

The most distinct features are observed in the intracellular and extracellular loops (Figure 3). Differences in the conformation of loops were rationalized, as illustrated by ICL2:

- ICL2 is helical in human Adenosine-2A receptor (hA2AAR) and tB1AR but is coil-like in the three other templates (Figure 4). In the former two structures an Arg sidechain in TMH4 forms a hydrogen bond with a mainchain carbonyl atom of the ICL2 helices, capping the helix C-termini (Figure 4A&B). Additionally, a Tyr sidechain within the ICL2 helices forms a hydrogen bond with the Asp residue of the DRY motif in TMH5, perhaps further helping to stabilise these loop structures.
- Although hB2AR also has a basic polar residue (Lys) at the corresponding position to the Arg residues and forms a hydrogen bond to a mainchain carbonyl group in ICL2, its shorter length may not be sufficient to stabilise the loop in a helical conformation (Figure 4C); the distance between the donor and acceptor atoms is somewhat longer in hB2AR (3.36 Å) compared with tB1AR (2.40 Å) and hA2AAR (3.07 Å). Furthermore, the Asp of the DRY motif forms a hydrogen bond with a Ser and not the corresponding Tyr of ICL2. In fact, molecular dynamics simulations have suggested that hB2AR is also able to adopt a helical ICL2 conformation and form the ionic lock in the inactive state and that this inactive conformational equilibrium in hB2AR may form the basis for the differential basal activity observed relative to tB1AR and hAA2AR [36]. Dror et al suggested that differences in ICL2 helix stability may underlie this difference in basal activity [36]; we propose that the lack of the helix-capping Arg residue in hB2AR and the presence of a Lys residue instead may provide such a basis for differences in ICL2 helix stability. For the purpose of our study we have used the conformation observed in the crystal structure.
- sRHO has a coil-like ICL2 and even though it does have an Arg residue at the corresponding position to those in hAA2AR and tB1AR, the Arg seems unable to form a hydrogen bond with the backbone of ICL2 due to repulsion by a Lys sidechain. Additionally, there is no polar sidechain to interact with the Asp of the DRY motif.
- bRHO has a coil-like ICL2 and has neither a capping helix C-termini interaction nor a hydrogen bond between the Glu of the ERY motif and ECL2.

Therefore, we suggest that the ICL2 helical structures observed in hAA2AR and tB1AR are indicated by the presence of a Tyr at position 156, the absence of a basic sidechain at position 159 and an Arg at position 164 in the MSA (Figure 4); the occurrence of these residues in target GPCRs would require either hAA2AR or tB1AR to be used for modelling this loop. For instance, using these criteria Melanocortin receptor 4 (hMC4R) and Cannabinoid receptor 2 (hCNR2) are predicted to have helical ICL2 conformations. In hMC4R the predicted helix-capping Arg in TMH4 has been associated with morbid obesity when mutated (R165W) and functional experiments have shown that this mutation reduces receptor activation [37]. Further work has shown that this loss in activity is likely due to reduced expression at the cell membrane [38]. Loss of the helix-capping interaction in R165W may affect the correct trafficking of this receptor, providing a possible mechanism for the observed malfunction of this mutant.

In the next step of our analysis, our intention was to identify which of the structural features in Table 5 are present in our set of 14 target GPCRs by comparing the amino acid sequences in the MSA (Figure S1) and tallying the results for each target GPCR (Text S2). However, some features such as the presence of secondary structure within loops and helix extensions could not be determined from sequence comparisons alone (indicated by a ‘?’ in the tables within Text S2).

Similarity to the extensions of helix 5 and 6 in sRHO was assessed by calculating the sequence similarity (Tables S4 and S5). See Figure S1 for the sequence regions used for these calculations. Where a target GPCR shows highest sequence similarity to sRHO (and not bRHO) and the sequence similarity is > = 50% then we suggest that sRHO should be used as a template for TMHs 5 and 6. However, it should be noted that helix 5 becomes extended in opsin compared to rhodopsin [23], indicating a structure-function relationship rather than a sequence-structure relationship. Therefore the sequence similarity results can serve only as guiding information as to the existence of extended TMHs 5 and 6.

### The contribution of structural features to receptor conformation

**Figure 5:**
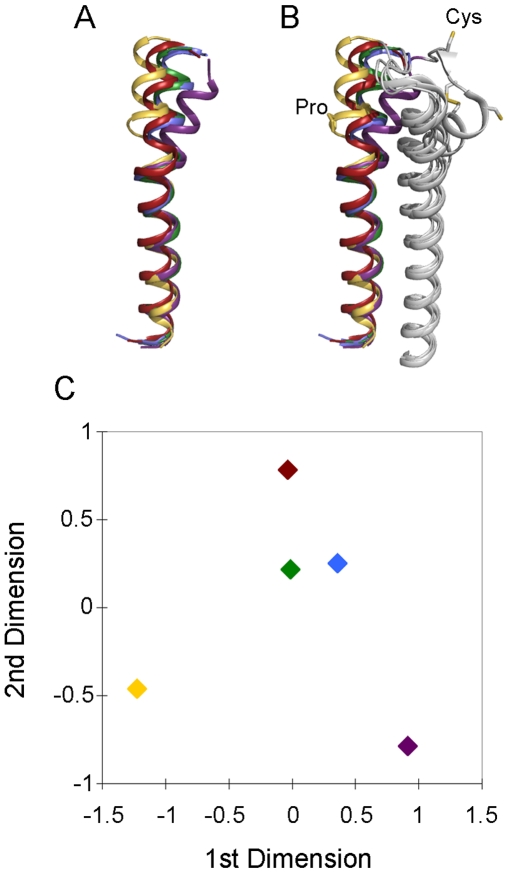
Structural diversity of TMH2 in the five template GPCRs. A) tB1AR (blue) hB2AR (green) and bRHO (red) have similar conformations, with hAA2AR (purple) and sRHO (yellow) diverging at the extracellular end. B) sRHO has an insertion (Pro) relative to the other structures, which causes a bulge in the helix. The kink in hAA2AR may be accentuated due to the presence of a Cys residue forming a disulphide bridge with ECL2. C) Multidimensional scaling of the distances between TMH2 of the five template structures (distance is measured by RMSD [Table 6]). The stress value was 0.09. Colouring is the same as in A and B.

It is unclear which of the features summarised in Table 5 have a large effect on receptor function and overall structure and which have moderate effects. Therefore in order to assess the impact of these features on the template structures, the root mean squared deviation (RMSD) was calculated between each TMH of each of the 5 templates; Table 6 shows a sample of these results (TMH2), with the remaining TMH RMSDs being found in Text S3). In the case of TMH2, it is clear that the insertion in sRHO relative to the other four templates and the disulphide bridge between ECL1 and ECL2 make a larger contribution to the structural diversity of TMH2 than the Gly-Gly bulge in bRHO (Figure 5 and Table 5).

**Table 6:** The RMSD of residues in TMH2.

	hAA2AR	tB1AR	hB2AR	sRHO	bRHO
**hAA2AR**	0.00	2.19	2.10	2.65	2.93
**tB1AR**	2.19	0.00	0.61	2.65	3.57
**hB2AR**	2.10	0.61	0.00	2.57	2.81
**sRHO**	2.65	2.65	2.57	0.00	3.57
**bRHO**	2.93	3.57	2.81	3.57	0.00

### Integration of results into workflow for comparative modelling template selection

**Figure 6:**
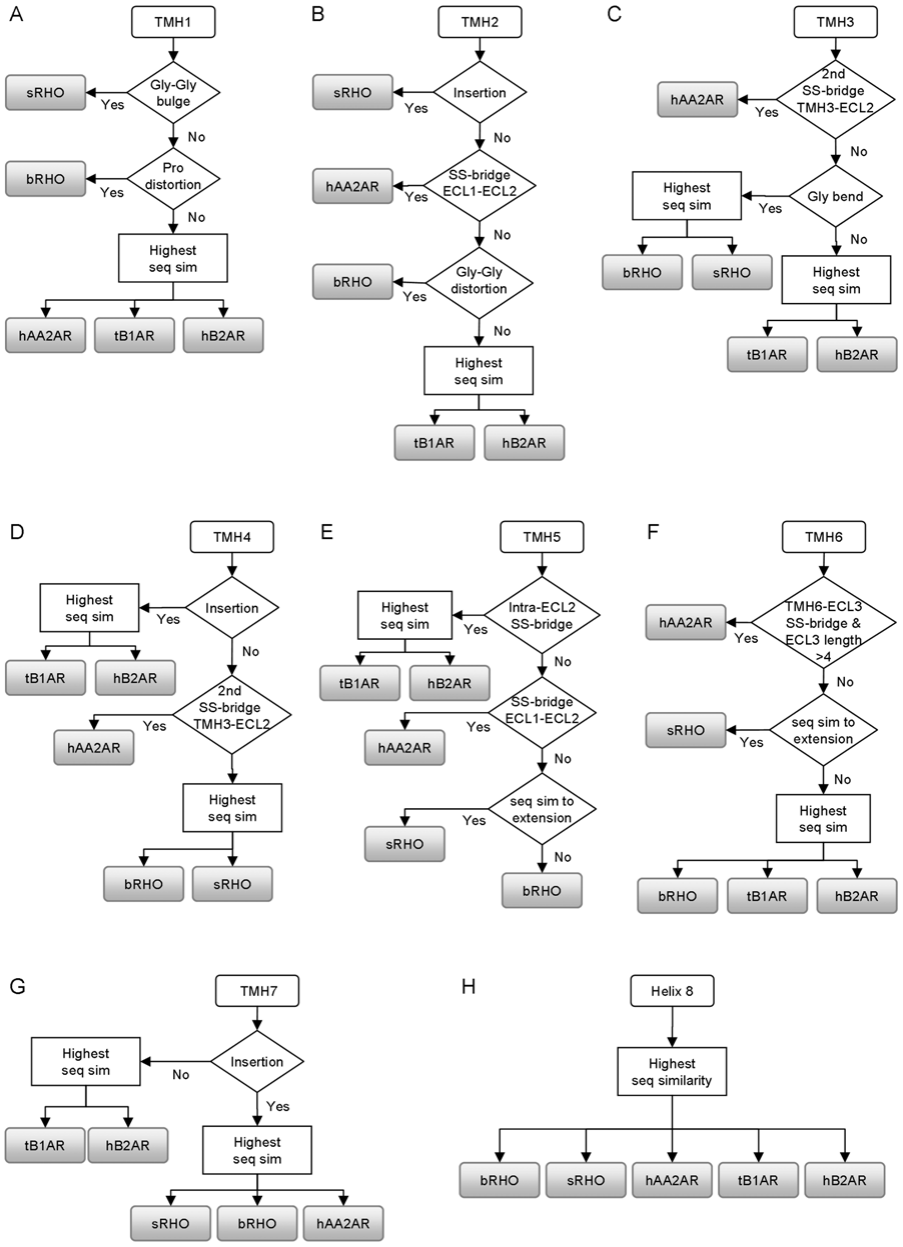
Integrated workflows for template selection. Shows the decision process for selecting which template should be used for modelling A) TMH1 B) TMH2 C) TMH3 D) TMH4 E) TMH5 F) TMH6 G) TMH7 and H) helix 8. For each helix the presence of particular features in a target GPCR are identified using a multiple sequence alignment with the five template GPCRs. Structural features include: a Gly-Gly bulge/distortion, a Pro distortion, insertions, disulphide bridges (SS-bridges), a Gly bend and sequence similarity to the helix extensions of sRHO. Where a target GPCR does not have any of the features then a template is chosen based on the sequence similarity score (seq sim).

We have integrated all the analyses (sequence similarity scores, structural features and RMSD calculations) to develop workflows for the selection of templates for homology model building of each of the seven TMHs and helix 8 (Figure 6). For example Figure 6B shows the suggested template selection workflow for TMH2 and is essentially a formalization of the results detailed in Table 4, Table 6 and Figure 5. Using these workflow schemes, we suggest the most suitable template to use for modelling each TMH and helix 8 of the 14 target GPCRs (Table 7). In some instances, more than one template is suggested by the workflows. In these cases we have selected one (shown as italic in Table 7) based on either similarity to a flanking TMH, higher resolution or to optimize the space between helices (i.e. avoid clashes or narrow gaps). Our analysis suggests that multiple templates should be used for homology model building of 13 of the 14 target GPCRs (human Rhodopsin (hRHO) is the exception due to its extremely high sequence similarity with bRHO). We also observe that for certain TMHs, particular template GPCRs are suggested for modelling most of the target GPCRs e.g. for TMHs 4 and 5 sRHO or bRHO are suggested for all cases, due mainly to the fact that none of the 14 target GPCRs have structural features that are observed in tB1AR, hB2AR or hAA2AR. TMH5 of hAA2AR, tB1AR and hB2AR superimpose relatively well, except for the extracellular portion, where hAA2AR diverges from the adrenergic structures. We propose that the difference observed in TMH5 of hAA2AR relative to the adrenergic structures is due to constrictions imposed by the conformation of ECL2 in these three structures, a key indicator of which is the presence of particular disulphide bridges. As none of the 14 target GPCRs has Cys residues at the ECL2 disulphide bridge positions, then either bRHO or sRHO are predicted to be the best templates. Of course, when building homology models using the combinations of TMHs shown in Table 7, the templates need to be superimposed first (e.g. using the seven highly conserved residues as reference points). By doing so, the orientation of the helices relative to one another is maintained.

**Table 7:** The template suggestions for each of the 14 target GPCRs.

Target GPCR ID	TMH1	TMH2	TMH3	TMH4	TMH5	TMH6	TMH7	H8
**hRHO**	bRHO	bRHO	bRHO	bRHO	bRHO	bRHO	bRHO	bRHO
**hACM1**	hB2AR	hB2AR	tB1AR	sRHO	sRHO	sRHO	hAA2AR	hAA2AR
**hDRD2**	hB2AR	tB1AR	tB1AR	sRHO	bRHO	sRHO	*hAA2AR* bRHO	hAA2AR
**hV1AR**	*hAA2AR* tB1AR	sRHO	tB1AR	sRHO	bRHO	tB1AR	hAA2AR *bRHO*	sRHO
**hV2R**	hB2AR	sRHO	tB1AR *hB2AR*	sRHO	bRHO	tB1AR	hAA2AR	bRHO
**hCCR5**	tB1AR	tB1AR	tB1AR	bRHO	bRHO	tB1AR	hAA2AR	bRHO
**hMC4R**	hAA2AR	hB2AR	tB1AR	sRHO	bRHO	hAA2AR	hAA2AR	hAA2AR
**hCNR1**	tB1AR	sRHO	tB1AR	sRHO	bRHO	tB1AR	hAA2AR	hAA2AR
**hCNR2**	tB1AR	sRHO	tB1AR	sRHO	bRHO	tB1AR	hAA2AR *sRHO*	bRHO
**hP2RY1**	hAA2AR	tB1AR	tB1AR	sRHO *bRHO*	bRHO	tB1AR	hAA2AR *sRHO*	sRHO
**hP2RY12**	hB2AR	tB1AR	tB1AR	sRHO	bRHO	tB1AR*hB2AR*	hAA2AR	bRHO
**hFSHR**	tB1AR	hB2AR	tB1AR *hB2AR*	sRHO	bRHO	hB2AR	sRHO	hAA2AR
**hLHCGR**	tB1AR	hB2AR	tB1AR *hB2AR*	sRHO	bRHO	hB2AR	sRHO	hAA2AR
**hTSHR**	tB1AR	hB2AR	hB2AR	sRHO	bRHO	hB2AR	sRHO	hAA2AR

Where two templates are suggested by a workflow, the preferential choice is shown in italics.

### Modelling the intracellular and extracellular loops

The loop regions of GPCRs tend to be less conserved than the TMH regions and in some cases are structurally diverse in the available GPCR structures e.g. ECL2. Therefore, comparative modelling of these loop regions presents a more difficult task than for the TMHs. In fact, it is not possible to use any of the five GPCR structures to model loops in the targets when:

1. Structural data are unavailable (e.g. ICL3 is missing in hAA2AR and hB2AR due to fusion with T4 lysozyme).
2. A target differs in length to all the available template structures. ICL3 is the most extreme example, being more than 100 residues long in Muscarinic acetylcholine receptor M1 (hACM1) and Dopamine D2 receptor (hDRD2).
3. A target has a similar length to an available template structure but it is missing a structural feature e.g. the TMH6-ECL3 disulphide bridge in hAA2AR.

In all of these three cases it will be necessary to use fragment-search based methods [39], [40] or *ab initio* based methods [35] for predicting these loop conformations. Indeed it has already been demonstrated that a more accurate model of the binding pocket and better docking of the ligand was achieved for hB2AR when ECL2 was built *ab initio* rather than using bRHO as a template [41].

For most of the 14 targets, ICL1 and ECL1 can be modelled with reasonable confidence, due to the similarity in length and conformation. In such cases, the template prediction for the flanking TMHs should be used to guide the loop template selection. The presence of certain conformations e.g. helical, β-strand etc can be predicted by particular amino acids. For instance, the helices observed in ECL2 of tB1AR and hB2AR are probably constrained by the intra-ECL2 disulphide bridge and the β-strand structure observed in hAA2AR is probably constrained by a disulphide bridge between ECL1 and ECL2. Therefore similarly positioned cysteines in a template would indicate that the adrenergic structures or adenosine structure should be used to model ECL2. Alternatively, where a template is not able to form either of these disulphide bridges and where sequence similarity to the rhodopsin structures is observed alongside experimental evidence of a β-hairpin conformation of ECL2, then we suggest that ECL2 should be built as a β-hairpin using rhodopsin as a template. For instance, a sheet-like fold of ECL2 and its general localization between the transmembrane helices in C-C chemokine receptor type 5 (CCR5) is consistent with results concerning different accessibility of two antibodies versus the two different strands of the sheet [42]–[44]. However, where neither the potential disulphide bridge forming cysteines are observed nor a β-hairpin conformation implicated, then we suggest that ECL2 be modelled *de novo*.

Additionally, where available, experimental data can be used to constrain the conformation of loops. For example:

1. There is evidence that a disulphide bridge is present in ECL3 of MC4R [45] and
2. The NMR solution structure of ICL3 in Vasopressin V2 receptor (hV2R) was recently published [46], negating the requirement for modelling this portion of the receptor.

Therefore, the decision of how best to model the intracellular and extracellular loops needs to be done on a case-by-case basis. Our suggestions are detailed in Table S6.

### Identification of conserved water molecules stabilizing GPCR structure

Water molecules can have important roles in stabilizing protein structure and therefore where possible, buried water molecules that form stabilizing interactions in template structures should be incorporated into homology models before minimization.

Campillo and colleagues performed an analysis of water molecules in the vicinity of highly conserved amino acids in three crystal structures of bovine rhodopsin [47]. They identified six water molecules that were present in all three crystal structures and that were in the environment of certain conserved amino acids, speculating that these water molecules are likely to be present throughout the rhodopsin family of GPCRs.

In fact, we find that only four of these water molecules are also observed in any of the other four template structures (Table S7).

The first of these water molecules (P6.50) is located in a small cavity between TMHs 6 and 7, stabilizing the Pro induced distortion of TMH6 and linking TMHs 6 and 7. This water molecule is observed in all of the template structures except tB1AR, indicating a conserved role in stabilizing GPCR structures (it should be noted that tB1AR has the lowest resolution of all the five template structures).

The second conserved water molecule is observed in hB2AR, sRHO and bRHO, located close to the Pro induced kink of TMH7. In all three of these structures this water molecule forms a hydrogen bond to the mainchain amide group of the highly conserved N7.49 as well as to the sidechain of the highly conserved D2.50.

Similar to the previous water molecule, the third conserved water molecule is observed in hB2AR, sRHO and bRHO and is located close to the Pro induced kink of TMH7. In all three of these structures this water molecule forms a hydrogen bond to the sidechain of the highly conserved N7.49 and the sidechain of the highly conserved D2.50 therefore linking TMH2 and 7.

The fourth conserved water molecule is observed in all of the templates except hAA2AR, although the network of interactions varies from structure to structure. However, in all four structures there is a water molecule that forms a hydrogen bond to the sidechain of the highly conserved W6.48 and either directly to mainchain or sidechain atoms groups in TMH7 or indirectly via a network of water-mediated hydrogen bonds (sRHO).

It appears that each of these four conserved water molecules has a role in linking TMHs and stabilizing helix distortions. The role of these waters in signal transduction is discussed by Angel et al [48]. Therefore, it is suggested that these particular water molecules should be incorporated when building homology models of GPCRs, as recently demonstrated by a MC4R model where functional data were consistent with the interaction sites of the water molecules [49].

## Discussion

In this work we have carried out extensive sequence and structural comparative analyses of the available crystal structures of GPCRs. These analyses have allowed us to identify particular residues, motifs, or intra-molecular interactions that serve as predictors for the presence of certain structural features observed in the crystal structures. We have incorporated these predictors into a workflow for identifying which of the template structures should be used for building homology models of a set of GPCRs of unknown structure. We have shown that the decision of which single template to use when building a homology model of a GPCR of unknown structure is not straightforward. It has been shown previously that in the absence of an established modelling protocol, serious flaws are observed in structural models of GPCRs [50]. This work provides the first comprehensive analysis of currently available GPCR structures for aiding the selection of templates for GPCR homology modelling. Our analyses show that in general, multiple templates should be selected, based upon the presence or absence of structural features in TMHs or loops.

### Structural features are better predictors than sequence similarity

If sequence similarity of the entire serpentine domain (the region from the start of TMH1 to the end of TMH7) and helix 8 is used as the sole criteria for homology modelling template selection then a template may be selected that lacks a particular functionally important structural feature. For instance, hACM1 is most similar to tB1AR and hB2AR across the entire serpentine domain and helix 8 (Table 1). However, using either of the two adrenergic structures to build a homology model of hACM1 would result in TMH5 and 6 not being built with the predicted extensions. Likewise, the template structure may contain structural features that the target GPCR does not contain, in which case a feature may be introduced that does not exist in the GPCR of interest. For instance, across the entire serpentine domain and helix 8, human P2Y purinoreceptor 12 (hP2RY12) is most similar to tB1AR (**Table 3**). However, using the tB1AR structure to build a homology model of hP2RY12 would result in a helical conformation for ICL2, whereas in fact it is unlikely to be so due to the lack of particular polar sidechains that constrain this loop in a helical conformation in hAA2AR and tB1AR (the Arg in TMH4 that is observed to cap helical ICL2 and the Tyr that interacts with the Asp/Glu of the (D/E)RY motif). These examples illustrate that particular structural features can be better predictors of overall GPCR structure than sequence similarity. Comparison of Table 7 and Figure S2 further highlights the TMHs of the 14 target GPCRs that are poorly predicted by sequence similarity alone.

### Conserved proline distortions in the TMHs complicate GPCR homology modeling

There are multiple target GPCRs that do not have a Pro at a corresponding position to the Pro distortions observed in TMHs 2 and 5 of the template GPCRs (either the target does not have a Pro at all or the Pro is in a shifted position relative to all of the five templates). Therefore, there is the possibility that TMH distortions may be incorrectly introduced into a model. In fact, structural and evolutionary analysis of the Pro pattern of TMH2 in family A GPCRs suggests that an insertion/deletion has led to two different (bulged or kinked) structures for TMH2 that are indicated by the relative position of the Pro in a MSA [28]. Where a Pro is shifted in a target GPCR relative to the template GPCRs, the helix distortion will also be shifted and therefore this will require careful manipulation. Where a Pro is missing in a target GPCR, it might be assumed that the distortion of the TMH should be removed. However, studies have indicated that although mutation to a Pro in a TMH initially induces a kink, further mutations act to stabilize the kink through packing interactions, at which point the Pro is no longer required to maintain the kink [51], [52]. Therefore, we speculate that even though particular target GPCRs do not have a Pro in TMH2 or 5 like in the template GPCRs, they may still have a vestigial non-Pro kink. In light of this, we suggest that when modelling these non-Pro containing target GPCRs both kinked and non-kinked helices should be considered and assessed on a case-by-case basis using mutagenesis data.

### Directing future structural studies of GPCRs

For some particular portions of the 14 target GPCRs, it will not be possible to model the structure through homology to the five templates (see entries marked ‘-’ in Table S6). The identification of these non-homologous regions demonstrates how our analyses can be used to improve structural knowledge in the future. It would be sensible for future crystallization studies to focus on those GPCRs that contain unique features not observed in current experimental structures e.g. the extremely large ICL3 observed in hACM1 and hDRD2 or where uncertainty exists about TMH distortions due to lack of a Pro in particular GPCRs. It is highly likely that there are other conformations of ECL2 apart from the three observed in the five templates e.g. neither hCNR1 nor hCNR2 have the conserved cysteines that form a disulphide bridge between ECL2 and TMH3 in most family A GPCRs. Careful consideration of the “uniqueness” of GPCRs relative to the five templates before selecting one for crystallization studies could help to increase the novelty and impact of newly acquired structural data. Where the identified “unique” features are shared with other GPCRs of unknown 3D structure then an experimental structure will provide valuable information for building homology models of these related GPCRs.

### Opsin versus rhodopsin structure

Our analysis relates only to template selection for modelling the inactive conformation of GPCRs. The publication of the crystal structure of opsin bound to the extreme C-terminal segment of the alpha subunit of transducin provides the opportunity for building comparative models of GPCRs in a (partially) active state [23]. Although it has recently been demonstrated that an inactive structure of hB2AR can be used to retrieve agonists and antagonists through virtual screening [53], the availability of an active GPCR structure is an exciting development for pharmacological research of GPCRs as an active (or even partially active) conformation of these receptors adds valuable information for structure-based drug design and mechanistic studies. However, it remains to be seen whether the mechanisms underlying GPCR activation are similar throughout this superfamily. Perhaps the repertoire of activated GPCR conformations is more diverse than currently observed for inactive GPCR conformations. For instance, experimental evidence suggests that there are conformational differences between active GPCR structures with respect to the activating ligand [54] or the interacting G-protein subtype [55], [56]. The partially active opsin structure may also be suitable for comparative modelling of basally active GPCRs [57]. However, the structural basis of basal activity in some GPCRs may actually be rather less distinct than the tilting and restructuring of helices observed in opsin compared to rhodopsin; the difference in basal activity between hB2AR (high) and tB1AR (low) has been attributed to lack of helical structure in ICL2 in the former, resulting in altered interactions with the DRY motif [21] and perhaps to Gα [36]. The question of whether opsin is a reliable template for modelling activated and basally active GPCRs is therefore open to discussion and is likely to remain so until additional crystal structures of active GPCRs emerge.

We have performed a rigorous and systematic analysis of the available experimental GPCR structures, identifying common, different and unique sequence and structural motifs that can be used to guide template selection for homology modelling. Our analysis indicates that in general, the structural features of target GPCRs cannot be captured using only one of the experimental GPCR structures as a template for homology modelling. Consequently, we suggest that the use of multiple templates when building comparative models of GPCRs is likely to lead to more accurate results. Indeed, a recent study demonstrated that automated modelling of human neurokinin-1 (NK1) receptor was enriched by a factor of 2.6 when a combination of bRHO and hB2AR were used to construct models rather than when used as single templates [58]. The recent blind assessment of methods for GPCR structure modelling revealed that the best predictions relied on homology modelling approaches and that progress in the GPCR homology model building field will require improvements in the current prediction methods to “add value” to the best available templates [59]. The mutagenesis data stored in GPCR databases such as the SSFA [60], GRIS [61] and GPCRDB [62] provide a means of verifying homology models through identification of structure-function relationships of particular sidechains [58], [63]. We believe that our analysis of the recently solved GPCR structures contributes to a more consistent method for GPCR template selection that opens new ways to fundamentally improve the quality of GPCR homology model building.

## Materials and Methods

### Dataset

The amino acid sequences and three dimensional structures of the template GPCRs used for analysis were obtained from the Protein Data Bank (http://www.rcsb.org/pdb) [64]. Where more than one experimental structure was available for a particular GPCR the structure with the highest resolution was used. Where more than one chain was found in a PDB file, the longest chain appearing first in the file was chosen for further analysis. A set of 14 target GPCRs was constructed whereby each member is found in humans, has been shown to be associated with a particular disease and has no experimentally determined structure. Receptors were chosen so that each of the four main phylogenetic groups of GPCR family A were represented in our target dataset (including the most populated cluster within each group) [34]. The sequences of the fourteen target GPCRs were downloaded from UniProt (http://www.uniprot.org) [65].

### Superimposition of template structures

The template structures were superimposed using Sybyl 8.0 (Tripos Inc., St. Louise, Missouri, 63144, USA). The highly conserved residues found within each transmembrane helix (as defined by the Ballesteros-Weinstein nomenclature [66]) were used as the reference points for structural superimposition of backbone atoms.

### Defining the boundaries of the seven transmembrane helices and helix eight

We first identified the boundaries of each of these helices in each of the template structures. This was achieved by looking at the hydrogen bonds formed between mainchain atom groups within the structure. The N-terminal boundary of a helix was defined as the first residue of a helix to form an intra-helical mainchain-mainchain hydrogen bond via its mainchain carbonyl atom group. The C-terminal boundary of a helix was defined as the last residue of a helix to form an intra-helical mainchain-mainchain hydrogen bond via its mainchain amide atom group.

### Sequence alignment

The multiple sequence alignment (MSA) of the template and target GPCR sequences was produced using a two tier approach. Firstly, ClustalW was used to create an automatic alignment of all of the template and target GPCR sequences [67]. Then the MSA was manually refined, taking into account the structural superimposition of the templates.

### Sequence similarity calculations

Pairwise sequence similarity calculations were performed between each template sequence and each target sequence. Due to the variation within the extracellular and intracellular loop regions, we restricted the similarity analysis to the seven TMHs and helix eight. For each of these helices, we set the leftmost boundary (i.e. the start position) as that of the template whose helix starts last in the MSA and the rightmost boundary (i.e. the end position) as that of the template whose helix ends first in the MSA.

In some instances the amino acid sequence of the crystal structure differs from the corresponding wild-type sequence. In those cases where the GPCR was fused to T4 lysozyme at ICL3 (hAA2AR; hB2AR), the T4 lysozyme sequence was removed. Where point mutations were introduced into a GPCR, the mutant residue type was used in the sequence alignment rather than the wild-type residue.

The percentage sequence similarity (PSS) between two sequences was calculated by:
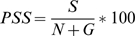
(1)Where S is the number of similar positions (defined by a BLOSUM62 matrix score of >0 [68]), *N* is the number of aligned positions and *G* is the number of internal gap positions.

### Structural similarity analyses

The RMSD of the TMH backbone atoms was calculated for each pair of template structures using the McLachlan algorithm [69] as implemented in the program ProFit (Martin, A.C.R., http://www.bioinf.org.uk/software/profit/). For each TMH we set the N-terminal boundary (i.e. start position) as that of the template whose helix starts last in the structural superposition and the C-terminal end (i.e. end position) as that of the template whose helix ends first in the structural superposition.

### Identification of unique structural features in template GPCRs

The superimposed structures were compared manually to identify differences (structural features) that could be incorporated into our modelling workflow assessment. We considered features such as helix kinks and bulges [70], [71], extension of helices [17], disulphide bridges [72], [73] and the conformation and secondary structure of loops [24], [74].

## Supporting Information

Table S1 The RMSD of residues in the common helical regions after superimposition using the seven conserved residues.
(0.03 MB DOC)

Table S2 The PDB residues identified as forming the seven TMHs and helix 8 in the five template structures.
(0.03 MB DOC)

Table S3 The RMSD of residues in the common helical regions after superimposition using these same residues.
(0.03 MB DOC)

Table S4 Sequence similarity scores between each template and target GPCR for TMH5 intracellular extension.
(0.05 MB DOC)

Table S5 Sequence similarity scores between each template and target GPCR for TMH6 intracellular extension.
(0.05 MB DOC)

Table S6 The template suggestions for the seven transmembrane helices and three intracellular and three extracellular loops of the 14 target GPCRs.
(0.07 MB DOC)

Table S7 Conserved water molecules observed in the five template structures.
(0.03 MB DOC)

Figure S1 The multiple sequence alignment of five template and 14 target GPCRs.
(0.05 MB PDF)

Figure S2 The highest sequence similarity templates for each of the TMHs and helix 8.
(0.55 MB PDF)

Text S1 The sequence similarity scores between the five template structures and each of the 14 target GPCRs for TMH1, TMH3-7 and helix 8.
(0.22 MB DOC)

Text S2 The prediction of structural features present in the five template structures in the 14 target GPCRs.
(0.84 MB DOC)

Text S3 The root mean squared deviation between each of the five template GPCR structures for TMH1, TMH3-7 and helix 8.
(0.08 MB DOC)
