# Supplementary material for: Comparative Sequence and Structural Analyses of G-Protein-Coupled Receptor Crystal Structures and Implications for Molecular Models
Source: PLoS One. 2009 Sep 16;4(9):e7011. doi: 10.1371/journal.pone.0007011 (PMC2738427; doi:10.1371/journal.pone.0007011)
Supplement: Table S1 — The RMSD of residues in the common helical regions after superimposition using the seven conserved residues. (0.03 MB DOC) [file pone.0007011.s001.doc]

Table S1: The RMSD of residues in the common helical regions after superimposition using the seven conserved residues.

|  | **hAA2AR** | **tB1AR** | **hB2AR** | **sRHO** | **bRHO** |
| --- | --- | --- | --- | --- | --- |
| **hAA2AR** | 0.00 | 2.33 | 2.25 | 3.10 | 3.21 |
| **tB1AR** | 2.33 | 0.00 | 0.63 | 2.96 | 3.17 |
| **hB2AR** | 2.25 | 0.63 | 0.00 | 2.82 | 3.05 |
| **sRHO** | 3.10 | 2.96 | 2.82 | 0.00 | 4.03 |
| **bRHO** | 3.21 | 3.17 | 3.05 | 4.03 | 0.00 |
