# Supplementary material for: Comparative Sequence and Structural Analyses of G-Protein-Coupled Receptor Crystal Structures and Implications for Molecular Models
Source: PLoS One. 2009 Sep 16;4(9):e7011. doi: 10.1371/journal.pone.0007011 (PMC2738427; doi:10.1371/journal.pone.0007011)
Supplement: Table S2 — The PDB residues identified as forming the seven TMHs and helix 8 in the five template structures. (0.03 MB DOC) [file pone.0007011.s002.doc]

Table S2: The PDB residues identified as forming the seven TMHs and helix 8 in the five template structures.

|  | **TMH1** | **TMH2** | **TMH3** | **TMH4** | **TMH5** | **TMH6** | **TMH7** | **H8** |
| --- | --- | --- | --- | --- | --- | --- | --- | --- |
| **hAA2AR** | 6-34 | 39-69 | 73-107 | 117-142 | 173-206 | 222-259 | 266-292 | 292-306 |
| **tB1AR** | 39-69 | 74-105 | 111-145 | 154-180 | 204-238 | 284-316 | 321-344 | 346-357 |
| **hB2AR** | 30-61 | 66-97 | 102-137 | 146-172 | 196-230 | 266-299 | 304-329 | 329-341 |
| **sRHO** | 30-62 | 67-99 | 104-139 | 148-173 | 194-239 | 244-287 | 293-319 | 319-330 |
| **bRHO** | 33-65 | 70-101 | 105-140 | 149-173 | 200-226 | 241-278 | 284-309 | 310-323 |
|  |  |  |  |  |  |  |  |  |
