# Supplementary material for: Comparative Sequence and Structural Analyses of G-Protein-Coupled Receptor Crystal Structures and Implications for Molecular Models
Source: PLoS One. 2009 Sep 16;4(9):e7011. doi: 10.1371/journal.pone.0007011 (PMC2738427; doi:10.1371/journal.pone.0007011)
Supplement: Table S3 — The RMSD of residues in the common helical regions after superimposition using these same residues. (0.03 MB DOC) [file pone.0007011.s003.doc]

Table S3: The RMSD of residues in the common helical regions after superimposition using these same residues.

|  | **hAA2AR** | **tB1AR** | **hB2AR** | **sRHO** | **bRHO** |
| --- | --- | --- | --- | --- | --- |
| **hAA2AR** | 0.00 | 2.19 | 2.10 | 2.65 | 2.93 |
| **tB1AR** | 2.19 | 0.00 | 0.61 | 2.65 | 3.57 |
| **hB2AR** | 2.10 | 0.61 | 0.00 | 2.57 | 2.81 |
| **sRHO** | 2.65 | 2.65 | 2.57 | 0.00 | 3.57 |
| **bRHO** | 2.93 | 3.57 | 2.81 | 3.57 | 0.00 |
