# Supplementary material for: Comparative Sequence and Structural Analyses of G-Protein-Coupled Receptor Crystal Structures and Implications for Molecular Models
Source: PLoS One. 2009 Sep 16;4(9):e7011. doi: 10.1371/journal.pone.0007011 (PMC2738427; doi:10.1371/journal.pone.0007011)
Supplement: Table S4 — Sequence similarity scores between each template and target GPCR for TMH5 intracellular extension. (0.05 MB DOC) [file pone.0007011.s004.doc]

Table S4: Sequence similarity scores between each template and target GPCR for TMH5 intracellular extension.

|  | **hAA2AR** | **tB1AR** | **hB2AR** | **sRHO** | **bRHO** |
| --- | --- | --- | --- | --- | --- |
| **hRHO** | 0.00 | 0.00 | 0.00 | 0.00 | **100.00** |
| **hACM1** | 0.00 | 0.00 | 0.00 | **66.00** | 11.00 |
| **hDRD2** | 0.00 | 0.00 | 0.00 | 22.00 | 0.00 |
| **hV1AR** | **33.00** | **33.00** | **33.00** | 11.00 | 0.00 |
| **hV2R** | **11.00** | **11.00** | **11.00** | **11.00** | 0.00 |
| **hCCR5** | 0.00 | 0.00 | 0.00 | **11.00** | 0.00 |
| **hMC4R** | 14.00 | 14.00 | 14.00 | **44.00** | 0.00 |
| **hCNR1** | 0.00 | 0.00 | 0.00 | **33.00** | 11.00 |
| **hCNR2** | 0.00 | 0.00 | 0.00 | **22.00** | 0.00 |
| **hP2RY1** | 0.00 | 0.00 | 0.00 | **22.00** | 0.00 |
| **hP2RY12** | 0.00 | 0.00 | 0.00 | 0.00 | 0.00 |
| **hFSHR** | 0.00 | 0.00 | 0.00 | **11.00** | 0.00 |
| **hLHCGR** | 0.00 | 0.00 | 0.00 | **11.00** | 0.00 |
| **hTSHR** | 0.00 | 0.00 | 0.00 | **11.00** | 0.00 |
|  |  |  |  |  |  |

The highest scoring template(s) are indicated by bold, underlined font.
