# Supplementary material for: Comparative Sequence and Structural Analyses of G-Protein-Coupled Receptor Crystal Structures and Implications for Molecular Models
Source: PLoS One. 2009 Sep 16;4(9):e7011. doi: 10.1371/journal.pone.0007011 (PMC2738427; doi:10.1371/journal.pone.0007011)
Supplement: Table S6 — The template suggestions for the seven transmembrane helices and three intracellular and three extracellular loops of the 14 target GPCRs. (0.07 MB DOC) [file pone.0007011.s006.doc]

**Table S6:** The template suggestions for the seven transmembrane helices and three intracelluar and extracellular loops of the 14 target GPCRs**.**

| **Target GPCR ID** | **TMH1** | **ICL1** | **TMH2** | **ECL1** | **TMH3** | **ICL2** | **TMH4** | **ECL2** | **TMH5** | **ICL3** | **TMH6** | **ECL3** | **TMH7** | **H8** |
| --- | --- | --- | --- | --- | --- | --- | --- | --- | --- | --- | --- | --- | --- | --- |
| **hRHO** | bRHO | bRHO | bRHO | bRHO | bRHO | bRHO | bRHO | bRHO | bRHO | bRHO | bRHO | bRHO | bRHO | bRHO |
| **hACM1** | hB2AR | hB2AR | hB2AR | hB2AR | tB1AR | sRHO | sRHO | sRHO / - | sRHO | - | sRHO | - | hAA2AR | hAA2AR |
| **hDRD2** | hB2AR | sRHO | tB1AR | tB1AR | tB1AR | sRHO | sRHO | - | bRHO | - | sRHO | tB1AR | hAA2AR  bRHO | hAA2AR |
| **hV1AR** | hAA2AR  tB1AR | hAA2AR | sRHO | - | tB1AR | sRHO | sRHO | bRHO / - | bRHO | - | tB1AR | - | hAA2AR  bRHO | sRHO |
| **hV2R** | hB2AR | - | sRHO | - | tB1AR hB2AR | sRHO | sRHO | bRHO / - | bRHO | * | tB1AR | - | hAA2AR | bRHO |
| **hCCR5** | tB1AR | tB1AR | tB1AR | tB1AR | tB1AR | sRHO | bRHO | bRHO | bRHO | - | tB1AR | - | hAA2AR | bRHO |
| **hMC4R** | hAA2AR | hAA2AR | hB2AR | - | tB1AR | tB1AR | sRHO | - | bRHO | bRHO/ - | hAA2AR | - & SS-bridge | hAA2AR | hAA2AR |
| **hCNR1** | tB1AR | sRHO | sRHO | - | tB1AR | sRHO | sRHO | - | bRHO | - | tB1AR | - | hAA2AR | hAA2AR |
| **hCNR2** | tB1AR | sRHO | sRHO | - | tB1AR | tB1AR | sRHO | - | bRHO | - | tB1AR | - | hAA2AR  sRHO | bRHO |
| **hP2RY1** | hAA2AR | tB1AR | tB1AR | tB1AR | tB1AR | sRHO | sRHO bRHO | bRHO | bRHO | - | tB1AR | - | hAA2AR  sRHO | sRHO |
| **hP2RY12** | hB2AR | - | tB1AR | tB1AR | tB1AR | sRHO | sRHO | bRHO | bRHO | - | tB1AR  hB2AR | - | hAA2AR | bRHO |
| **hFSHR** | tB1AR | hB2AR | hB2AR | - | tB1AR hB2AR | sRHO | sRHO | - | bRHO | - | hB2AR | sRHO | sRHO | hAA2AR |
| **hLHCGR** | tB1AR | hB2AR | hB2AR | - | tB1AR hB2AR | sRHO | sRHO | - | bRHO | - | hB2AR | sRHO | sRHO | hAA2AR |
| **hTSHR** | tB1AR | hB2AR | hB2AR | - | hB2AR | sRHO | sRHO | - | bRHO | - | hB2AR | sRHO | sRHO | hAA2AR |

- indicates that it is not possible to use any of the five templates for homology modeling

Where two templates are suggested by a workflow, the preferential choice is underlined.

SS-bridge indicates that a disulphide bridge should be built.

* NMR solution structure available
