# Supplementary material for: Comparative Sequence and Structural Analyses of G-Protein-Coupled Receptor Crystal Structures and Implications for Molecular Models
Source: PLoS One. 2009 Sep 16;4(9):e7011. doi: 10.1371/journal.pone.0007011 (PMC2738427; doi:10.1371/journal.pone.0007011)
Supplement: Table S7 — Conserved water molecules observed in the five template structures. (0.03 MB DOC) [file pone.0007011.s007.doc]

**Table S7: Conserved water molecules observed in the five template structures.**

|  | **Water 1** | **Water 2** | **Water 3** | **Water 4** |
| --- | --- | --- | --- | --- |
| hAA2AR | 502 | - | - | - |
| tB1AR | - | - | - | 2007 |
| hB2AR (B) | 529 | 532 | 548 | 534 |
| sRHO | 510 | 506 | 504 | 501 |
| bRHO | 964 | 2017 | 2030 | 2015 |

Chain ID (in brackets) and sequence number of the water molecules as found in the PDB file are given.
