# Supplementary material for: Comparative Sequence and Structural Analyses of G-Protein-Coupled Receptor Crystal Structures and Implications for Molecular Models
Source: PLoS One. 2009 Sep 16;4(9):e7011. doi: 10.1371/journal.pone.0007011 (PMC2738427; doi:10.1371/journal.pone.0007011)
Supplement: Figure S1 — The multiple sequence alignment of five template and 14 target GPCRs. (0.05 MB PDF) [file pone.0007011.s008.pdf]

```
                                                             TMH1              ICL1             TMH2
                                                                  1.50                          2.50
hAA2AR_3EML  ---------------------------------IMGSSVYITVELAIAVLAILGNVLVCWAVWL--NSNLQNVTNYFVVSLAAADIAVGVLAIP-FAITI
tB1AR_2VT4   ----------------------------------QWEAGMSLLMALVVLLIVAGNVLVIAAIGS--TQRLQTLTNLFITSLACADLVVGLLVVP-FGATL
hB2AR_2RH1   --------------------------------DEVWVVGMGIVMSLIVLAIVFGNVLVITAIAK--FERLQTVTNYFITSLACADLVMGLAVVP-FGAAH
sRHO_2Z73    -----------ETWWYNPSIVVHPHWREFDQVPDAVYYSLGIFIGICGIIGCGGNGIVIYLFTK--TKSLQTPANMFIINLAFSDFTFSLVNGFPLMTIS
bRHO_1U19    MNGTEGPNFYVPFSNKTGVVRSPFEAPQYYLAEPWQFSMLAAYMFLLIMLGFPINFLTLYVTVQ--HKKLRTPLNYILLNLAVADLFMVFGGFT-TTLYT
hRHO         MNGTEGPNFYVPFSNATGVVRSPFEYPQYYLAEPWQFSMLAAYMFLLIVLGFPINFLTLYVTVQ--HKKLRTPLNYILLNLAVADLFMVLGGFT-STLYT
hACM1        ------------MNTSAPPAVSPNITVLAPGKGPWQVAFIGITTGLLSLATVTGNLLVLISFKV--NTELKTVNNYFLLSLACADLIIGTFSMN-LYTTY
hDRD2        ---MDPLNLSWYDDDLERQNWSRPFNGSDGKADRPHYNYYATLLTLLIAVIVFGNVLVCMAVSR--EKALQTTTNYLIVSLAVADLLVATLVMP-WVVYL
hV1AR        SSPWWPLATGAGNTSREAEALGEGNGPPRDVRNEELAKLEIAVLAVTFAVAVLGNSSVLLALHR--TPRKTSRMHLFIRHLSLADLAVAFFQVLPQMCWD
hV2R         MLMASTTSAVPGHPSLPSLPSNSSQERPLDTRDPLLARAELALLSIVFVAVALSNGLVLAALARRGRRGHWAPIHVFIGHLCLADLAVALFQVLPQLAWK
hCCR5        -------MDYQVSSPIYDINYYTSEPCQKINVKQIAARLLPPLYSLVFIFGFVGNMLVILILIN--CKRLKSMTDIYLLNLAISDLFF-LLTVP-FWAHY
hMC4R        GMHTSLHLWNRSSYRLHSNASESLGKGYSDGGCYEQLFVSPEVFVTLGVISLLENILVIVAIAK--NKNLHSPMYFFICSLAVADMLVSVSNGS-ETIVI
hCNR1        EFYNKSLSSFKENEENIQCGENFMDIECFMVLNPSQQLAIAVLSLTLGTFTVLENLLVLCVILH-SRSLRCRPSYHFIGSLAVADLLGSVIFVYSFIDFH
hCNR2        ----MEECWVTEIANGSKDGLDSNPMKDYMILSGPQKTAVAVLCTLLGLLSALENVAVLYLILS-SHQLRRKPSYLFIGSLAGADFLASVVFACSFVNFH
hP2RY1       AAFLAGPGSSWGNSTVASTAAVSSSFKCALTKTGFQFYYLPAVYILVFIIGFLGNSVAIWMFVF--HMKPWSGISVYMFNLALADFLYVLTLPA-LIFYY
hP2Y12       ------------MQAVDNLTSAPGNTSLCTRDYKITQVLFPLLYTVLFFVGLITNGLAMRIFFQ--IR-SKSNFIIFLKNTVISDLLMILTFPF-KILSD
hFSHR        FDMTYTEFDYDLCNEVVDVTCSPKPDAFNPCEDIMGYNILRVLIWFISILAITGNIIVLVILTT-SQYKL-TVPRFLMCNLAFADLCIGIYLLL-IASVD
hLHCGR       SELSGWDYEYGFCL-PKTPRCAPEPDAFNPCEDIMGYDFLRVLIWLINILAIMGNMTVLFVLLT-SRYKL-TVPRFLMCNLSFADFCMGLYLLL-IASVD
hTSHR        LQAFDSHYDYTICGDSEDMVCTPKSDEFNPCEDIMGYKFLRIVVWFVSLLALLGNVFVLLILLT-SHYKL-NVPRFLMCNLAFADFCMGMYLLL-IASVD

                  ECL1                   TMH3               ICL2             TMH4              ECL2
                                                         3.50                       4.50
hAA2AR_3EML  ST-G---------FCAACHGCLFIACFVLVLTQSSIFSLLAIAIDRYIAIRIPLRYNGLVTGTRAKGIIAICWVLSFAIGL-TPMLGWNNCGQ/-----S
tB1AR_2VT4   VVRG--------TWLWGSFLCELWTSLDVLCVTASIETLCVIAIDRYLAITSPFRYQSLMTRARAKVIICTVWAISALVSFLPIMMHWWRD---EDPQAL
hB2AR_2RH1   ILMK--------MWTFGNFWCEFWTSIDVLCVTASIETLCVIAVDRYFAITSPFKYQSLLTKNKARVIILMVWIVSGLTSFLPIQMHWYRA---THQEAI
sRHO_2Z73    CFLK--------KWIFGFAACKVYGFIGGIFGFMSIMTMAMISIDRYNVIGRPMAASKKMSHRRAFIMIIFVWLWSVLWAI-GPIFGWGAY---TLEGVL
bRHO_1U19    SLHG--------YFVFGPTGCNLEGFFATLGGEIALWSLVVLAIERYVVVCKPM-SNFRFGENHAIMGVAFTWVMALACAA-PPLVGWSRY---IPEGMQ
hRHO         SLHG--------YFVFGPTGCNLEGFFATLGGEIALWSLVVLAIERYVVVCKPM-SNFRFGENHAIMGVAFTWVMALACAA-PPLAGWSRY---IPEGLQ
hACM1        LLMG--------HWALGTLACDLWLALDYVASNASVMNLLLISFDRYFSVTRPLSYRAKRTPRRAALMIGLAWLVSFVLWA-PAILFWQYL---VGERTV
hDRD2        EVVG--------EWKFSRIHCDIFVTLDVMMCTASILNLCAISIDRYTAVAMPMLYNTRYSKRRVTVMISIVWVLSFTISC-PLLFGLNNA---------
hV1AR        ITY---------RFRGPDWLCRVVKHLQVFGMFASAYMLVVMTADRYIAVCHPLKTLQQ-PARRSRLMIAAAWVLSFVLST-PQYFVFSMI---EVNNVT
hV2R         ATD---------RFRGPDALCRAVKYLQMVGMYASSYMILAMTLDRHRAICRPMLAYRHGSGAHWNRPVLVAWAFSLLLSL-PQLFIFAQR---NVEGGS
hCCR5        AAA---------QWDFGNTMCQLLTGLYFIGFFSGIFFIILLTIDRYLAVVHAVFALKARTVTFGVVTSVITWVVAVFASL-PGIIFTRSQ---KEGL--
hMC4R        TLLNSTDTDAQS---FTVNIDNVIDSVICSSLLASICSLLSIAVDRYFTIFYALQYHNIMTVKRVGIIISCIWAACTVSGI-LFIIYS------------
hCNR1        VFHR----------KDSRNVFLFKLGGVTASFTASVGSLFLTAIDRYISIHRPLAYKRIVTRPKAVVAFCLMWTIAIVIAV-LPLLGWNCE---------
hCNR2        VFHG----------VDSKAVFLLKIGSVTMTFTASVGSLLLTAIDRYLCLRYPPSYKALLTRGRALVTLGIMWVLSALVSY-LPLMGWTCC---------
hP2RY1       FNKT--------DWIFGDAMCKLQRFIFHVNLYGSILFLTCISAHRYSGVVYPLKSLGRLKKKNAICISVLVWLIVVVAIS-PILFYSGTG---VRKNKT
hP2Y12       AKLG--------TGPLRTFVCQVTSVIFYFTMYISISFLGLITIDRYQKTTRPFKTSNPKNLLGAKILSVVIWAFMFLLSL-PNMILTNRQ---PRDKNV
hFSHR        IHTKSQYHNYAIDW-QTGAGCDAAGFFTVFASELSVYTLTAITLERWHTITHAMQLDCKVQLRHAASVMVMGWIFAFAAAL-FPIFGISSY---MKVSI-
hLHCGR       SQTKGQYYNHAIDW-QTGSGCSTAGFFTVFASELSVYTLTVITLERWHTITYAIHLDQKLRLRHAILIMLGGWLFSSLIAM-LPLVGVSNY---MKVSI-
hTSHR        LYTHSEYYNHAIDW-QTGPGCNTAGFFTVFASELSVYTLTVITLERWYAITFAMRLDRKIRLRHACAIMVGGWVCCFLLAL-LPLVGISSY---AKVSI-
```

```
                   ECL2                        TMH5                                          ICL3
                                                  5.50
hAA2AR_3EML  QGCGEGQVACLFEDV-----VPMNYMVYFNFFACVLVPLLLMLGVYLRIFLAARRQL/------------------------------------------
tB1AR_2VT4   KCYQDPGC-CDF--------VTNRAYAIASSIISFYIPLLIMIFVALRVYREAKEQI/------------------------------------------
hB2AR_2RH1   NCYAEETC-CDF--------FTNQAYAIASSIVSFYVPLVIMVFVYSRVFQEAKRQL/------------------------------------------
sRHO_2Z73    -------CNCSFDYIS--RDSTTRSNILCMFILGFFGPILIIFFCYFNIVMSVSNHEKEMAAMAKRLNA-------------------------------
bRHO_1U19    -------CSCGIDYYTPHEETNNESFVIYMFVVHFIIPLIVIFFCYGQLVFTVKEAAA------------------------------------------
hRHO         -------CSCGIDYYTLKPEVNNESFVIYMFVVHFTIPMIIIFFCYGQLVFTVKEAAA------------------------------------------
hACM1        -----LAGQCYIQF------LSQPIITFGTAMAAFYLPVTVMCTLYWRIYRETENRARELAALQGSETPGKGGGSSSSSERSQPGAEGSPETPPGRCCRC
hDRD2        -----DQNECII---------ANPAFVVYSSIVSFYVPFIVTLLVYIKIYIVLRRRRKRVNTKRSSRAFRAHLRAPLKGNCTHPEDMKLCTVIMKSNGSF
hV1AR        -----KARDCWATFI---QPWGSRAYVTWMTGGIFVAPVVILGTCYGFICYNIWCNVRG-----------------------------------------
hV2R         -----GVTDCWACFA---EPWGRRTYVTWIALMVFVAPTLGIAACQVLIFREIHASLVPGPSERPGGRRRGRRT--------------------------
hCCR5        ------HYTCSSHFPYSQYQFWKNFQTLKIVILGLVLPLLVMVICYSGILKTLLRCRNE-----------------------------------------
hMC4R        ----------------------DSSAVIICLITMFFTMLALMASLYVHMFLMARLHIKRIAVL-------------------------------------
hCNR1        --KLQSVCSD----------IFPHIDETYLMFWIGVTSVLLLFIVYAYMYILWKAHSHAVRMIQRGTQKSIIIHTSEDG---------------------
hCNR2        ----PRPCSE----------LFPLIPNDYLLSWLLFIAFLFSGIIYTYGHVLWKAHQHVASLS--GHQ--------------------------------
hP2RY1       -------ITCYDTT-SDEYLRSYFIYSMCTTVAMFCVPLVLILGCYGLIVRALIYKDLD-----------------------------------------
hP2Y12       -------KKCSFLK-SEFG-LVWHEIVNYICQVIFWINFLIVIVCYTLITKELYRSYVR-----------------------------------------
hFSHR        ---------CLPMDI----DSPLSQLYVMSLLVLNVLAFVVICGCYIHIYLTVRNPNIVSS---------------------------------------
hLHCGR       ---------CFPMDV----ETTLSQVYILTILILNVVAFFIICACYIKIYFAVRNPELMAT---------------------------------------
hTSHR        ---------CLPMDT----ETPLALAYIVFVLTLNIVAFVIVCCCYVKIYITVRNPQYNPG---------------------------------------

                                                        ICL3
hAA2AR_3EML  --------------------------------------------------------------------------------------------------RS
tB1AR_2VT4   ----------------------------------------------------------------------------------------------------
hB2AR_2RH1   ---------------------------------------------------------------------------------------------------K
sRHO_2Z73    --------------------------------------------------------------------------------------------KELRKAQA
bRHO_1U19    ---------------------------------------------------------------------------------------------QQQESAT
hRHO         ---------------------------------------------------------------------------------------------QQQESAT
hACM1        CRAPRLLQAYSWKEEEEEEDEGSMESLTS------SEGEEPGSEVVIKMPMVDPEAQAPTKQPPRSSPNTVKRPTKKGRDRAGKGQKPRGKEQLAKRKTF
hDRD2        PVNRRRVEAARRAQELEMEMLSSTSPPERTRYSPIPPSHHQLTLPDPSHHGLHSTPDSPAKPEKNGHAKDHPKIAKIFEIQTMPNGKTRTSLKTMSRRKL
hV1AR        --------------------------------------------------------------------KTASRQSKGAEQAGVAFQKGFLLAPCVSSVKS
hV2R         --------------------------------------------------------------------------------------------GSPGEGAH
hCCR5        ----------------------------------------------------------------------------------------------------
hMC4R        ----------------------------------------------------------------------------------------------PGTGAI
hCNR1        --------------------------------------------------------------------------------------------KVQVTRPD
hCNR2        --------------------------------------------------------------------------------------------DRQVPGMA
hP2RY1       ----------------------------------------------------------------------------------------------------
hP2Y12       ------------------------------------------------------------------------------------------------TRGV
hFSHR        ----------------------------------------------------------------------------------------------------
hLHCGR       ----------------------------------------------------------------------------------------------------
hTSHR        ----------------------------------------------------------------------------------------------------
```

```
                              TMH6                        ECL3              TMH7              Helix 8
                                        6.50                                       7.50
hAA2AR_3EML  TLQKEVHAAKSLAIIVGLFALCWLPLHIINCFTFFCPDCSHA---------PLWLMYLAIVLSHTNSVVNPFIYAYRIREFRQTFRKIIRSHVLRQ----
tB1AR_2VT4   ---REHKALKTLGIIMGVFTLCWLPFFLVNIVNVFNRD--LV---------PDWLFVAFNWLGYANSAMNPIIYC-RSPDFRKAFKRLLA----------
hB2AR_2RH1   FCLKEHKALKTLGIIMGTFTLCWLPFFIVNIVHVIQDN--LI---------RKEVYILLNWIGYVNSGFNPLIYC-RSPDFRIAFQELLCL---------
sRHO_2Z73    GANAEMRLAKISIVIVSQFLLSWSPYAVVALLAQFGPLE-WV---------TPYAAQLPVMFAKASAIHNPMIYSVSHPKFREAISQTFPWVLTCCQFDD
bRHO_1U19    TQKAEKEVTRMVIIMVIAFLICWLPYAGVAFYIFTHQGS-DF---------GPIFMTIPAFFAKTSAVYNPVIYIMMNKQFRNCMVTTLCCGKNPLGDDE
hRHO         TQKAEKEVTRMVIIMVIAFLICWVPYASVAFYIFTHQGS-NF---------GPIFMTIPAFFAKSAAIYNPVIYIMMNKQFRNCMLTTICCGKNPLGDDE
hACM1        SLVKEKKAARTLSAILLAFILTWTPYNIMVLVSTFCKDCV-----------PETLWELGYWLCYVNSTINPMCYALCNKAFRDTFRLLLLCRWDKRRWRK
hDRD2        SQQKEKKATQMLAIVLGVFIICWLPFFITHILNIHC-DC-NI---------PPVLYSAFTWLGYVNSAVNPIIYTTFNIEFRKAFLKILHC---------
hV1AR        ISRAKIRTVKMTFVIVTAYIVCWAPFFIIQMWSVWDPMSVWTES-------ENPTITITALLGSLNSCCNPWIYMFFSGHLLQDCVQSFPCCQNMKEKFN
hV2R         VSAAVAKTVRMTLVIVVVYVLCWAPFFLVQLWAAWDPEAPL----------EGAPFVLLMLLASLNSCTNPWIYASFSSSVSSELRSLLCCARGRTPPSL
hCCR5        --KKRHRAVRLIFTIMIVYFLFWAPYNIVLLLNTFQEF--FGLNNC-SSSRLDQAMQVTETLGMTHCCINPIIYAFVGEKFRNYLLVFFQKHIAKRFCKC
hMC4R        RQGANMKGAITLTILIGVFVVCWAPFFLHLIFYISCPQ--NPYCVC-----FMSHFNLYLILIMCNSIIDPLIYALRSQELRKTFKEIICCYPLGGLCDL
hCNR1        QARMDIRLAKTLVLILVVLIICWGPLLAIMVYDVFGKM--NKL--------IKTVFAFCSMLCLLNSTVNPIIYALRSKDLRHAFRSMFPSCEGTAQPLD
hCNR2        RMRLDVRLAKTLGLVLAVLLICWFPVLALMAHSLATTL--SDQ--------VKKAFAFCSMLCLINSMVNPVIYALRSGEIRSSAHHCLAHWKKCVRGLG
hP2RY1       NSPLRRKSIYLVIIVLTVFAVSYIPFHVMKTMNLRARLDFQTPAMC-AFNRVYATYQVTRGLASLNSCVDPILYFLAGDTFRRRLSRATRKASRRSEANL
hP2Y12       GKVPRKKVNVKVFIIIAVFFICFVPFHFARIPYTLSQT--RDVFDCTAENTLFYVKESTLWLTSLNACLDPFIYFFLCKSFRNSLISMLKCPNSATSLSQ
hFSHR        --SSDTRIAKRMAMLIFTDFLCMAPISFFAISASLKVP----------LITVSKAKILLVLFHPINSCANPFLYAIFTKNFRRDFFILLSKCGCYEMQAQ
hLHCGR       --NKDTKIAKKMAILIFTDFTCMAPISFFAISAAFKVP----------LITVTNSKVLLVLFYPINSCANPFLYAIFTKTFQRDFFLLLSKFGCCKRRAE
hTSHR        --DKDTKIAKRMAVLIFTDFICMAPISFYALSAILNKP----------LITVSNSKILLVLFYPLNSCANPFLYAIFTKAFQRDVFILLSKFGICKRQAQ

hAA2AR_3EML  -----------------
tB1AR_2VT4   -----------------
hB2AR_2RH1   -----------------
sRHO_2Z73    KETEDDKDAETEIPAGE
bRHO_1U19    ASTTVSKTETSQVAPA-
hRHO         ASATVSKTETSQVAPA-
hACM1        IPKRPGSVHRTPSRQC-
hDRD2        -----------------
hV1AR        KEDTDSMSRRQTFYSNN
hV2R         GPQDESCTTASSSLAKD
hCCR5        CSIFQQEAPERASSVYT
hMC4R        SSRY-------------
hCNR1        NSMGDSDCLHKHANNAA
hCNR2        SEAKEEAPRSSVTETEA
hP2RY1       QSKSEDMTLNILPEFKQ
hP2Y12       DNRKKEQDGGDPNEETP
hFSHR        IYRTETSSTVHNTHPRN
hLHCGR       LYRRKDFSAYTSNCKNG
hTSHR        AYRGQRVPPKNSTDIQV
```

Figure S1: The multiple sequence alignment of five template and twelve target GPCRs. Residues are coloured according to type: acidic (red), basic (blue), polar neutral (green), hydrophobic aliphatic (grey), hydrophobic aromatic (purple), special structural properties (brown) and disulphide bond former (orange). The transmembrane helix (TMH) regions used for sequence similarity calculations are shown in yellow, helix eight in grey and the extensions of TMH5 and 6 in turquoise. The Ballesteros-Weinstein nomenclature for each of the highly conserved residues found within each TMH are displayed. The intracellular loops and extracellular loops (ECL) are also indicated.
