# Supplementary material for: Comparative Sequence and Structural Analyses of G-Protein-Coupled Receptor Crystal Structures and Implications for Molecular Models
Source: PLoS One. 2009 Sep 16;4(9):e7011. doi: 10.1371/journal.pone.0007011 (PMC2738427; doi:10.1371/journal.pone.0007011)
Supplement: Figure S2 — The highest sequence similarity templates for each of the TMHs and helix 8. (0.55 MB PDF) [file pone.0007011.s009.pdf]

	TMH1	TMH2	TMH3	TMH4	TMH5	TMH6	TMH7	H8
hRHO	bRHO	bRHO	bRHO	bRHO	bRHO	bRHO	bRHO	bRHO
hACM1	hB2AR	hB2AR	tB1AR	hAA2AR	hB2AR	hAA2AR, tB1AR, sRHO	tB1AR, hB2AR	hAA2AR
hDRD2	hB2AR	tB1AR	tB1AR	tB1AR, hB2AR	tB1AR, hB2AR	tB1AR	tB1AR	tB1AR
hV1AR	hAA2AR, tB1AR	bRHO	tB1AR	tB1AR, hB2AR	bRHO	sRHO	hAA2AR, bRHO	sRHO
hV2R	hB2AR	hAA2AR, sRHO, bRHO	hAA2AR, sRHO	sRHO	bRHO	sRHO	hAA2AR	bRHO
hCCR5	tB1AR	hAA2AR, tB1AR	tB1AR	bRHO	bRHO	tB1AR	hAA2AR	bRHO
hMC4R	hAA2AR	bRHO	hAA2AR, tB1AR	sRHO	tB1AR, hB2AR, sRHO	tB1AR, hB2AR	hAA2AR	hAA2AR
hCNR1	tB1AR	hB2AR	hAA2AR	tB1AR	hAA2AR	hAA2AR, tB1AR, sRHO	hB2AR	tB1AR, hB2AR
hCNR2	tB1AR	sRHO	hAA2AR, tB1AR	tB1AR	hAA2AR, hB2AR	hAA2AR, tB1AR	hB2AR	tB1AR
hP2RY1	bRHO	sRHO	sRHO	hB2AR	hB2AR	tB1AR	hB2AR	sRHO
hP2Y12	hB2AR, sRHO	hAA2AR, tB1AR	tB1AR	tB1AR	bRHO	tB1AR, hB2AR	hAA2AR	bRHO
hFSHR	tB1AR	hB2AR	bRHO	sRHO	hAA2AR	sRHO	sRHO	tB1AR
hLHCGR	tB1AR	hB2AR	bRHO	sRHO	hAA2AR, bRHO	sRHO	sRHO	tB1AR
hTSHR	tB1AR	hB2AR	bRHO	sRHO	bRHO	hB2AR, sRHO	sRHO	tB1AR

Figure S2: The highest sequence similarity templates for each of the TMHs and helix 8. Each of the 14 target GPCRs (excepting hRHO) matches multiple templates across their entire length. In some cases, two or more of the templates are equally similar. These results indicate that sequence similarity alone is not a good indicator of which template to use for homology modeling. The templates are coloured using the following scheme: hAA2AR (purple), tB1AR (blue), hB2AR (green), sRHO (yellow) and bRHO (red).
