# Supplementary material for: Comparative Sequence and Structural Analyses of G-Protein-Coupled Receptor Crystal Structures and Implications for Molecular Models
Source: PLoS One. 2009 Sep 16;4(9):e7011. doi: 10.1371/journal.pone.0007011 (PMC2738427; doi:10.1371/journal.pone.0007011)
Supplement: Text S1 — The sequence similarity scores between the five template structures and each of the 14 target GPCRs for TMH1, TMH3-7 and helix 8. (0.22 MB DOC) [file pone.0007011.s010.doc]

Supporting Text S1

The sequence similarity scores between the five template structures and each of the 14 target GPCRs were calculated for each TMH and helix 8. All of the results (except those for TMH2 which are displayed in the accompanying paper) are shown below in Tables I to VII.

Table I: Sequence similarity scores between each template structure and each target GPCR for TMH1.

|  | **hAA2AR** | **tB1AR** | **hB2AR** | **sRHO** | **bRHO** |
| --- | --- | --- | --- | --- | --- |
| **hRHO** | 31.00 | 41.00 | 34.00 | 48.00 | **100.00** |
| **hACM1** | 44.00 | 48.00 | **55.00** | 41.00 | 31.00 |
| **hDRD2** | 51.00 | 55.00 | **62.00** | 34.00 | 37.00 |
| **hV1AR** | **44.00** | **44.00** | 41.00 | 31.00 | 27.00 |
| **hV2R** | 35.00 | 45.00 | **51.00** | 32.00 | 29.00 |
| **hCCR5** | 41.00 | **48.00** | 44.00 | 34.00 | 41.00 |
| **hMC4R** | **55.00** | 41.00 | 48.00 | 31.00 | 31.00 |
| **hCNR1** | 33.00 | **43.00** | 40.00 | 30.00 | 26.00 |
| **hCNR2** | 43.00 | **50.00** | 40.00 | 40.00 | 43.00 |
| **hP2RY1** | 37.00 | 34.00 | 31.00 | 48.00 | **55.00** |
| **hP2RY12** | 34.00 | 34.00 | **37.00** | **37.00** | 34.00 |
| **hFSHR** | 50.00 | **53.00** | 46.00 | 43.00 | 43.00 |
| **hLHCGR** | 43.00 | **53.00** | 46.00 | 43.00 | 43.00 |
| **hTSHR** | 46.00 | **50.00** | 43.00 | 36.00 | 40.00 |
|  |  |  |  |  |  |

The highest scoring template(s) are indicated by bold, underlined font.

Table II: Sequence similarity scores between each template structure and each target GPCR for TMH3.

|  | **hAA2AR** | **tB1AR** | **hB2AR** | **sRHO** | **bRHO** |
| --- | --- | --- | --- | --- | --- |
| **hRHO** | 54.00 | 45.00 | 40.00 | 60.00 | **100.00** |
| **hACM1** | 45.00 | **62.00** | 60.00 | 51.00 | 48.00 |
| **hDRD2** | 54.00 | **65.00** | 62.00 | 45.00 | 42.00 |
| **hV1AR** | 42.00 | **51.00** | 45.00 | 40.00 | 42.00 |
| **hV2R** | **48.00** | 45.00 | 45.00 | **48.00** | 45.00 |
| **hCCR5** | 45.00 | **57.00** | 48.00 | 45.00 | 48.00 |
| **hMC4R** | **48.00** | **48.00** | 45.00 | 37.00 | 40.00 |
| **hCNR1** | **48.00** | 45.00 | 42.00 | 34.00 | 42.00 |
| **hCNR2** | **48.00** | **48.00** | 40.00 | 40.00 | **48.00** |
| **hP2RY1** | 28.00 | 42.00 | 40.00 | **51.00** | 37.00 |
| **hP2RY12** | 28.00 | **54.00** | 45.00 | 48.00 | 37.00 |
| **hFSHR** | 51.00 | 40.00 | 40.00 | 48.00 | **54.00** |
| **hLHCGR** | 48.00 | 40.00 | 40.00 | 51.00 | **60.00** |
| **hTSHR** | 51.00 | 42.00 | 45.00 | 45.00 | **57.00** |
|  |  |  |  |  |  |

The highest scoring template(s) are indicated by bold, underlined font.

Table III: Sequence similarity scores between each template structure and each target GPCR for TMH4.

|  | **hAA2AR** | **tB1AR** | **hB2AR** | **sRHO** | **bRHO** |
| --- | --- | --- | --- | --- | --- |
| **hRHO** | 40.00 | 34.00 | 42.00 | 44.00 | **96.00** |
| **hACM1** | **56.00** | 53.00 | 53.00 | 48.00 | 44.00 |
| **hDRD2** | 52.00 | **57.00** | **57.00** | 52.00 | 44.00 |
| **hV1AR** | 48.00 | **50.00** | **50.00** | 48.00 | 40.00 |
| **hV2R** | 36.00 | 42.00 | 34.00 | **48.00** | 32.00 |
| **hCCR5** | 40.00 | 34.00 | 42.00 | 40.00 | **48.00** |
| **hMC4R** | 40.00 | 42.00 | 38.00 | **44.00** | 24.00 |
| **hCNR1** | 48.00 | **57.00** | 50.00 | 52.00 | 44.00 |
| **hCNR2** | 52.00 | **65.00** | 61.00 | 52.00 | 48.00 |
| **hP2RY1** | 28.00 | 38.00 | **46.00** | 44.00 | 44.00 |
| **hP2RY12** | 40.00 | **50.00** | 46.00 | 48.00 | 28.00 |
| **hFSHR** | 48.00 | 26.00 | 34.00 | **56.00** | 40.00 |
| **hLHCGR** | 44.00 | 46.00 | 42.00 | **60.00** | 48.00 |
| **hTSHR** | 44.00 | 34.00 | 34.00 | **48.00** | 36.00 |
|  |  |  |  |  |  |

The highest scoring template(s) are indicated by bold, underlined font.

Table IV: Sequence similarity scores between each template structure and each target GPCR for TMH5.

|  | **hAA2AR** | **tB1AR** | **hB2AR** | **sRHO** | **bRHO** |
| --- | --- | --- | --- | --- | --- |
| **hRHO** | 44.00 | 59.00 | 66.00 | 66.00 | **96.00** |
| **hACM1** | 44.00 | 51.00 | **55.00** | 37.00 | 37.00 |
| **hDRD2** | 48.00 | **74.00** | **74.00** | 44.00 | 59.00 |
| **hV1AR** | 37.00 | 44.00 | 48.00 | 51.00 | **62.00** |
| **hV2R** | 29.00 | 40.00 | 37.00 | 44.00 | **51.00** |
| **hCCR5** | 48.00 | 40.00 | 48.00 | 48.00 | **51.00** |
| **hMC4R** | 33.00 | **44.00** | **44.00** | **44.00** | 40.00 |
| **hCNR1** | **40.00** | 22.00 | 29.00 | 33.00 | 37.00 |
| **hCNR2** | **22.00** | 14.00 | **22.00** | 14.00 | 18.00 |
| **hP2RY1** | 40.00 | 55.00 | **59.00** | 44.00 | 51.00 |
| **hP2RY12** | 40.00 | 33.00 | 40.00 | 40.00 | **44.00** |
| **hFSHR** | **48.00** | 37.00 | 37.00 | 37.00 | 40.00 |
| **hLHCGR** | **40.00** | 37.00 | 37.00 | 29.00 | **40.00** |
| **hTSHR** | 37.00 | 33.00 | 33.00 | 29.00 | **40.00** |
|  |  |  |  |  |  |

The highest scoring template(s) are indicated by bold, underlined font.

Table V: Sequence similarity scores between each template structure and each target GPCR for TMH6.

|  | **hAA2AR** | **tB1AR** | **hB2AR** | **sRHO** | **bRHO** |
| --- | --- | --- | --- | --- | --- |
| **hRHO** | 45.00 | 48.00 | 45.00 | 54.00 | **96.00** |
| **hACM1** | **60.00** | **60.00** | 54.00 | **60.00** | 48.00 |
| **hDRD2** | 60.00 | **75.00** | 72.00 | 48.00 | 54.00 |
| **hV1AR** | 48.00 | 63.00 | 54.00 | **69.00** | 51.00 |
| **hV2R** | 42.00 | 57.00 | 48.00 | **63.00** | 54.00 |
| **hCCR5** | 45.00 | **63.00** | 60.00 | 48.00 | 36.00 |
| **hMC4R** | 51.00 | **54.00** | **54.00** | 48.00 | 45.00 |
| **hCNR1** | **54.00** | **54.00** | 45.00 | **54.00** | 48.00 |
| **hCNR2** | **51.00** | **51.00** | 45.00 | 48.00 | 42.00 |
| **hP2RY1** | 45.00 | **57.00** | 54.00 | 48.00 | 45.00 |
| **hP2RY12** | 36.00 | **45.00** | **45.00** | 36.00 | 42.00 |
| **hFSHR** | 36.00 | 33.00 | 42.00 | **48.00** | 39.00 |
| **hLHCGR** | 39.00 | 36.00 | 39.00 | **45.00** | 36.00 |
| **hTSHR** | 39.00 | 42.00 | **45.00** | **45.00** | 42.00 |
|  |  |  |  |  |  |

The highest scoring template(s) are indicated by bold, underlined font.

Table VI: Sequence similarity scores between each template and target GPCR for TMH7.

|  | **hAA2AR** | **tB1AR** | **hB2AR** | **sRHO** | **bRHO** |
| --- | --- | --- | --- | --- | --- |
| **hRHO** | 41.00 | 33.00 | 37.00 | 66.00 | **100.00** |
| **hACM1** | 50.00 | **58.00** | **58.00** | 37.00 | 33.00 |
| **hDRD2** | 45.00 | **75.00** | 58.00 | 37.00 | 45.00 |
| **hV1AR** | **45.00** | 37.00 | 41.00 | 33.00 | **45.00** |
| **hV2R** | **54.00** | 37.00 | 50.00 | 45.00 | 33.00 |
| **hCCR5** | **45.00** | 41.00 | 41.00 | 37.00 | 33.00 |
| **hMC4R** | **58.00** | 45.00 | 41.00 | 50.00 | 37.00 |
| **hCNR1** | 45.00 | 50.00 | **54.00** | 37.00 | 33.00 |
| **hCNR2** | 45.00 | 45.00 | **54.00** | 45.00 | 37.00 |
| **hP2RY1** | 41.00 | 41.00 | **45.00** | 41.00 | 37.00 |
| **hP2RY12** | **54.00** | 50.00 | 41.00 | 37.00 | 29.00 |
| **hFSHR** | 45.00 | 29.00 | 45.00 | **50.00** | 33.00 |
| **hLHCGR** | 45.00 | 29.00 | 41.00 | **50.00** | 33.00 |
| **hTSHR** | 45.00 | 29.00 | 41.00 | **50.00** | 33.00 |
|  |  |  |  |  |  |

The highest scoring template(s) are indicated by bold, underlined font.

Table VII: Sequence similarity scores between each template and target GPCR for helix 8.

|  | **hAA2AR** | **tB1AR** | **hB2AR** | **sRHO** | **bRHO** |
| --- | --- | --- | --- | --- | --- |
| **hRHO** | 41.00 | 33.00 | 33.00 | 50.00 | **100.00** |
| **hACM1** | **66.00** | 58.00 | 58.00 | 33.00 | 50.00 |
| **hDRD2** | 66.00 | **83.00** | 75.00 | 58.00 | 50.00 |
| **hV1AR** | 16.00 | 25.00 | 16.00 | **33.00** | 25.00 |
| **hV2R** | 25.00 | 33.00 | 33.00 | 8.00 | **41.00** |
| **hCCR5** | 25.00 | 16.00 | 16.00 | 41.00 | **58.00** |
| **hMC4R** | **83.00** | 66.00 | 66.00 | 33.00 | 41.00 |
| **hCNR1** | 50.00 | **58.00** | **58.00** | 25.00 | 41.00 |
| **hCNR2** | 33.00 | **41.00** | **41.00** | 25.00 | **41.00** |
| **hP2RY1** | 33.00 | 33.00 | 16.00 | **41.00** | 25.00 |
| **hP2RY12** | 50.00 | 41.00 | 41.00 | 33.00 | **66.00** |
| **hFSHR** | 58.00 | **66.00** | 58.00 | 16.00 | 33.00 |
| **hLHCGR** | **58.00** | **58.00** | 50.00 | 16.00 | 33.00 |
| **hTSHR** | **50.00** | **50.00** | 41.00 | 25.00 | 41.00 |
|  |  |  |  |  |  |

The highest scoring template(s) are indicated by bold, underlined font.
