# Supplementary material for: Comparative Sequence and Structural Analyses of G-Protein-Coupled Receptor Crystal Structures and Implications for Molecular Models
Source: PLoS One. 2009 Sep 16;4(9):e7011. doi: 10.1371/journal.pone.0007011 (PMC2738427; doi:10.1371/journal.pone.0007011)
Supplement: Text S2 — The prediction of structural features present in the five template structures in the 14 target GPCRs. (0.84 MB DOC) [file pone.0007011.s011.doc]

Supporting Text S2

The structural features identified in the five template structures (Table 5) were predicted to be present in the 14 target GPCRs by comparing the amino acid sequences in the multiple sequence alignment (Figure S1) – results shown below in Tables I to XIV.

**Table I**: Structural features predicted to be present in hRHO based on sequence comparison to the five template GPCRs.

|  |  | **hAA2AR** | **tB1AR** | **hB2AR** | **sRHO** | **bRHO** |
| --- | --- | --- | --- | --- | --- | --- |
| TMH1 | Pro distortion |  |  |  |  | + |
|  | Gly-Gly bulge |  |  |  | - |  |
| ICL1 | 3_10_ helix indicated by proton donor sidechain forming H-bond to acceptor group in helix 8. Followed by Leu. | - | - | - |  |  |
| TMH2 | Pro distortion | - | - | - | - |  |
|  | Gly-Gly distortion |  |  |  |  | + |
|  | Bulge due to insertion |  |  |  | - |  |
| ECL1 | Disulphide bridge to ECL2 | - |  |  |  |  |
|  | Beta-sheet structure (indicated by above disulphide bridge) | - |  |  |  |  |
| TMH3 | Conserved Cys forming disulphide bridge to ECL2 | + | + | + | + | + |
|  | Second disulphide bridge to ECL2 | - |  |  |  |  |
|  | Gly bend |  |  |  | + | + |
| ICL2 | Helix - Indicated by Arg in TMH4 and Tyr in ICL2. | - | - |  |  |  |
| TMH4 | Pro distortion | + | + | + | + | + |
|  | Bulge due to insertion |  | - | - |  |  |
| ECL2 | Disulphide bridge to TMH3 | + | + | + | + | + |
|  | Beta-sheet |  |  |  | + | + |
|  | Intra-ECL2 disulphide bridge |  | - | - |  |  |
|  | Alpha-helix – indicated by above disulphide bridge |  | - | - |  |  |
|  | Disulphide bridge to ECL1 | - |  |  |  |  |
|  | Beta-strand | - |  |  |  |  |
|  | Second disulphide bridge to TMH3 | - |  |  |  |  |
| TMH5 | Pro distortion | + | + | + | + | + |
|  | Helix extension |  |  |  | - |  |
| TMH6 | Pro distortion | + | + | + | + | + |
|  | Helix extension |  |  |  | - |  |
| ECL3 | TMH6-ECL3 disulphide bridge | - |  |  |  |  |
|  | 3_10_ helix – indicated by Glu sidechain forming H-bond to mainchain amide group (helix-capping). |  |  |  | - |  |
| TMH7 | Pro distortion | + | + | + | + | + |
| Helix 8 | Insertion | + |  |  | + | + |
|  |  |  |  |  |  |  |

+ indicates that hRHO has a structural feature that is observed in a particular template(s).

- indicates that hRHO does not have a structural feature that is observed in a particular template(s).

**Table II:** **Structural features predicted to be present in hACM1 based on sequence comparison to the five template GPCRs.**

|  |  | **hAA2AR** | **tB1AR** | **hB2AR** | **sRHO** | **bRHO** |
| --- | --- | --- | --- | --- | --- | --- |
| TMH1 | Pro distortion |  |  |  |  | - |
|  | Gly-Gly bulge |  |  |  | - |  |
| ICL1 | 3_10_ helix indicated by proton donor sidechain forming H-bond to acceptor group in helix 8. Followed by Leu. | - | - | - |  |  |
| TMH2 | Pro distortion | - | - | - | - |  |
|  | Gly-Gly distortion |  |  |  |  | - |
|  | Bulge due to insertion |  |  |  | - |  |
| ECL1 | Disulphide bridge to ECL2 | - |  |  |  |  |
|  | Beta-sheet structure (indicated by above disulphide bridge) | - |  |  |  |  |
| TMH3 | Conserved Cys forms disulphide bridge to ECL2 | + | + | + | + | + |
|  | Second disulphide bridge to ECL2 | - |  |  |  |  |
|  | Gly bend |  |  |  | - | - |
| ICL2 | Helix - Indicated by Arg in TMH4 and Tyr in ICL2. | + | + |  |  |  |
| TMH4 | Pro distortion | + | + | + | + | + |
|  | Bulge due to insertion |  | - | - |  |  |
| ECL2 | Disulphide bridge to TMH3 | + | + | + | + | + |
|  | Beta-sheet |  |  |  | ? | ? |
|  | Intra-ECL2 disulphide bridge |  | - | - |  |  |
|  | Alpha-helix – indicated by above disulphide bridge |  | - | - |  |  |
|  | Disulphide bridge to ECL1 | - |  |  |  |  |
|  | Beta-strand – indicated by above disulphide bridge | - |  |  |  |  |
|  | Second disulphide bridge to TMH3 | - |  |  |  |  |
| TMH5 | Pro distortion | + | + | + | + | + |
|  | Helix extension |  |  |  | ? |  |
| TMH6 | Pro distortion | + | + | + | + | + |
|  | Helix extension |  |  |  | ? |  |
| ECL3 | TMH6-ECL3 disulphide bridge | + |  |  |  |  |
|  | 3_10_ helix – indicated by Glu sidechain forming H-bond to mainchain amide group (helix-capping). |  |  |  | - |  |
| TMH7 | Pro distortion | + | + | + | + | + |
| Helix 8 | Insertion | + |  |  | + | + |
|  |  |  |  |  |  |  |

+ indicates that hACM1 has a structural feature that is observed in a particular template(s).

- indicates that hACM1 does not have a structural feature that is observed in a particular template(s)

? indicates that there is uncertainty about whether hACM1 has a structural feature that is observed in a particular template(s).

NB: ICL3 is extremely large compared to any template

**Table III: Structural features predicted to be present in hDRD2 based on sequence comparison to the five template GPCRs.**

|  |  | **hAA2AR** | **tB1AR** | **hB2AR** | **sRHO** | **bRHO** |
| --- | --- | --- | --- | --- | --- | --- |
| TMH1 | Pro distortion |  |  |  |  | - |
|  | Gly-Gly bulge |  |  |  | - |  |
| ICL1 | 3_10_ helix indicated by proton donor sidechain forming H-bond to acceptor group in helix 8. Followed by Leu. | - | - | - |  |  |
| TMH2 | Pro distortion | + | + | + | + |  |
|  | Gly-Gly distortion |  |  |  |  | - |
|  | Bulge due to insertion |  |  |  | - |  |
| ECL1 | Disulphide bridge to ECL2 | - |  |  |  |  |
|  | Beta-sheet structure (indicated by above disulphide bridge) | - |  |  |  |  |
| TMH3 | Conserved Cys forms disulphide bridge to ECL2 | + | + | + | + | + |
|  | Second disulphide bridge to ECL2 | - |  |  |  |  |
|  | Gly bend |  |  |  | - | - |
| ICL2 | Helix - Indicated by Arg in TMH4 and Tyr in ICL2. | + | + |  |  |  |
| TMH4 | Pro distortion | + | + | + | + | + |
|  | Bulge due to insertion |  | - | - |  |  |
| ECL2 | Disulphide bridge to TMH3 | + | + | + | + | + |
|  | Beta-sheet |  |  |  | ? | ? |
|  | Intra-ECL2 disulphide bridge |  | - | - |  |  |
|  | Alpha-helix – indicated by above disulphide bridge |  | - | - |  |  |
|  | Disulphide bridge to ECL1 | - |  |  |  |  |
|  | Beta-strand – indicated by above disulphide bridge | - |  |  |  |  |
|  | Second disulphide bridge to TMH3 | - |  |  |  |  |
| TMH5 | Pro distortion | + | + | + | + | + |
|  | Helix extension |  |  |  | ? |  |
| TMH6 | Pro distortion | + | + | + | + | + |
|  | Helix extension |  |  |  | ? |  |
| ECL3 | TMH6-ECL3 disulphide bridge | + |  |  |  |  |
|  | 3_10_ helix – indicated by Glu sidechain forming H-bond to mainchain amide group (helix-capping). |  |  |  | + |  |
| TMH7 | Pro distortion | + | + | + | + | + |
| Helix 8 | Insertion | + |  |  | + | + |
|  |  |  |  |  |  |  |

+ indicates that hDRD2 has a structural feature that is observed in a particular template(s).

- indicates that hDRD2 does not have a structural feature that is observed in a particular template(s)

? indicates that there is uncertainty about whether hDRD2 has a structural feature that is observed in a particular template(s).

**Table IV:** **Structural features predicted to be present in hV1AR based on sequence comparison to the five template GPCRs.**

|  |  | **hAA2AR** | **tB1AR** | **hB2AR** | **sRHO** | **bRHO** |
| --- | --- | --- | --- | --- | --- | --- |
| TMH1 | Pro distortion |  |  |  |  | - |
|  | Gly-Gly bulge |  |  |  | - |  |
| ICL1 | 3_10_ helix indicated by proton donor sidechain forming H-bond to acceptor group in helix 8. Followed by Leu. | + | + | + |  |  |
| TMH2 | Pro distortion | + | + | + | + |  |
|  | Gly-Gly distortion |  |  |  |  | - |
|  | Bulge due to insertion |  |  |  | + |  |
| ECL1 | Disulphide bridge to ECL2 | - |  |  |  |  |
|  | Beta-sheet structure (indicated by above disulphide bridge) | - |  |  |  |  |
| TMH3 | Conserved Cys forms disulphide bridge to ECL2 | + | + | + | + | + |
|  | Second disulphide bridge to ECL2 | - |  |  |  |  |
|  | Gly bend |  |  |  | ? | ? |
| ICL2 | Helix - Indicated by Arg in TMH4 and Tyr in ICL2. | + | + |  |  |  |
| TMH4 | Pro distortion | + | + | + | + | + |
|  | Bulge due to insertion |  | - | - |  |  |
| ECL2 | Disulphide bridge to TMH3 | + | + | + | + | + |
|  | Beta-sheet |  |  |  | ? | ? |
|  | Intra-ECL2 disulphide bridge |  | - | - |  |  |
|  | Alpha-helix – indicated by above disulphide bridge |  | - | - |  |  |
|  | Disulphide bridge to ECL1 | - |  |  |  |  |
|  | Beta-strand – indicated by above disulphide bridge | - |  |  |  |  |
|  | Second disulphide bridge to TMH3 | - |  |  |  |  |
| TMH5 | Pro distortion | + | + | + | + | + |
|  | Helix extension |  |  |  | - |  |
| TMH6 | Pro distortion | + | + | + | + | + |
|  | Helix extension |  |  |  | - |  |
| ECL3 | TMH6-ECL3 disulphide bridge | - |  |  |  |  |
|  | 3_10_ helix – indicated by Glu sidechain forming H-bond to mainchain amide group (helix-capping). |  |  |  | - |  |
| TMH7 | Pro distortion | + | + | + | + | + |
| Helix 8 | Insertion | + |  |  | + | + |
|  |  |  |  |  |  |  |

+ indicates that hV1AR has a structural feature that is observed in a particular template(s).

- indicates that hV1AR does not have a structural feature that is observed in a particular template(s)

? indicates that there is uncertainty about whether hV1AR has a structural feature that is observed in a particular template(s).

**Table V:** **Structural features predicted to be present in hV2R based on sequence comparison to the five template GPCRs.**

|  |  | **hAA2AR** | **tB1AR** | **hB2AR** | **sRHO** | **bRHO** |
| --- | --- | --- | --- | --- | --- | --- |
| TMH1 | Pro distortion |  |  |  |  | - |
|  | Gly-Gly bulge |  |  |  | - |  |
| ICL1 | 3_10_ helix indicated by proton donor sidechain forming H-bond to acceptor group in helix 8. Followed by Leu. | + | + | + |  |  |
| TMH2 | Pro distortion | + | + | + | + |  |
|  | Gly-Gly distortion |  |  |  |  | - |
|  | Bulge due to insertion |  |  |  | - |  |
| ECL1 | Disulphide bridge to ECL2 | - |  |  |  |  |
|  | Beta-sheet structure (indicated by above disulphide bridge) | - |  |  |  |  |
| TMH3 | Conserved Cys forms disulphide bridge to ECL2 | + | + | + | + | + |
|  | Second disulphide bridge to ECL2 | - |  |  |  |  |
|  | Gly bend |  |  |  | ? | ? |
| ICL2 | Helix - Indicated by Arg in TMH4 and Tyr in ICL2. | - | - |  |  |  |
| TMH4 | Pro distortion | + | + | + | + | + |
|  | Bulge due to insertion |  | - | - |  |  |
| ECL2 | Disulphide bridge to TMH3 | + | + | + | + | + |
|  | Beta-sheet |  |  |  | ? | ? |
|  | Intra-ECL2 disulphide bridge |  | - | - |  |  |
|  | Alpha-helix – indicated by above disulphide bridge |  | - | - |  |  |
|  | Disulphide bridge to ECL1 | - |  |  |  |  |
|  | Beta-strand – indicated by above disulphide bridge | - |  |  |  |  |
|  | Second disulphide bridge to TMH3 | - |  |  |  |  |
| TMH5 | Pro distortion | + | + | + | + | + |
|  | Helix extension |  |  |  | ? |  |
| TMH6 | Pro distortion | + | + | + | + | + |
|  | Helix extension |  |  |  | ? |  |
| ECL3 | TMH6-ECL3 disulphide bridge | - |  |  |  |  |
|  | 3_10_ helix – indicated by Glu sidechain forming H-bond to mainchain amide group (helix-capping). |  |  |  | ? |  |
| TMH7 | Pro distortion | + | + | + | + | + |
| Helix 8 | Insertion | + |  |  | + | + |
|  |  |  |  |  |  |  |

+ indicates that hV2R has a structural feature that is observed in a particular template(s).

- indicates that hV2R does not have a structural feature that is observed in a particular template(s)

? indicates that there is uncertainty about whether hV2R has a structural feature that is observed in a particular template(s).

**Table VI:** **Structural features predicted to be present in hCCR5 based on sequence comparison to the five template GPCRs.**

|  |  | **hAA2AR** | **tB1AR** | **hB2AR** | **sRHO** | **bRHO** |
| --- | --- | --- | --- | --- | --- | --- |
| TMH1 | Pro distortion |  |  |  |  | - |
|  | Gly-Gly bulge |  |  |  | - |  |
| ICL1 | 3_10_ helix indicated by proton donor sidechain forming H-bond to acceptor group in helix 8. Followed by Leu. | - | - | - |  |  |
| TMH2 | Pro distortion | + | + | + | + |  |
|  | Gly-Gly distortion |  |  |  |  | - |
|  | Bulge due to insertion |  |  |  | - |  |
| ECL1 | Disulphide bridge to ECL2 | - |  |  |  |  |
|  | Beta-sheet structure (indicated by above disulphide bridge) | - |  |  |  |  |
| TMH3 | Conserved Cys forms disulphide bridge to ECL2 | + | + | + | + | + |
|  | Second disulphide bridge to ECL2 | - |  |  |  |  |
|  | Gly bend |  |  |  | ? | ? |
| ICL2 | Helix - Indicated by Arg in TMH4 and Tyr in ICL2. | - | - |  |  |  |
| TMH4 | Pro distortion | + | + | + | + | + |
|  | Bulge due to insertion |  | - | - |  |  |
| ECL2 | Disulphide bridge to TMH3 | + | + | + | + | + |
|  | Beta-sheet |  |  |  | ? | ? |
|  | Intra-ECL2 disulphide bridge |  | - | - |  |  |
|  | Alpha-helix – indicated by above disulphide bridge |  | - | - |  |  |
|  | Disulphide bridge to ECL1 | - |  |  |  |  |
|  | Beta-strand | - |  |  |  |  |
|  | Second disulphide bridge to TMH3 | - |  |  |  |  |
| TMH5 | Pro distortion | + | + | + | + | + |
|  | Helix extension |  |  |  | - |  |
| TMH6 | Pro distortion | + | + | + | + | + |
|  | Helix extension |  |  |  | - |  |
| ECL3 | TMH6-ECL3 disulphide bridge | - |  |  |  |  |
|  | 3_10_ helix – indicated by Glu sidechain forming H-bond to mainchain amide group (helix-capping). |  |  |  | - |  |
| TMH7 | Pro distortion | + | + | + | + | + |
| Helix 8 | Insertion | + |  |  | + | + |
|  |  |  |  |  |  |  |

+ indicates that hCCR5has a structural feature that is observed in a particular template(s).

- indicates that hCCR5does not have a structural feature that is observed in a particular template(s)

? indicates that there is uncertainty about whether hCCR5has a structural feature that is observed in a particular template(s).

**Table VII:** **Structural features predicted to be present in hMC4R based on sequence comparison to the five template GPCRs.**

|  |  | **hAA2AR** | **tB1AR** | **hB2AR** | **sRHO** | **bRHO** |
| --- | --- | --- | --- | --- | --- | --- |
| TMH1 | Pro distortion |  |  |  |  | - |
|  | Gly-Gly bulge |  |  |  | - |  |
| ICL1 | 3_10_ helix indicated by proton donor sidechain forming H-bond to acceptor group in helix 8. Followed by Leu. | + | + | + |  |  |
| TMH2 | Pro distortion | - | - | - | - |  |
|  | Gly-Gly distortion |  |  |  |  | - |
|  | Bulge due to insertion |  |  |  | - |  |
| ECL1 | Disulphide bridge to ECL2 | - |  |  |  |  |
|  | Beta-sheet structure (indicated by above disulphide bridge) | - |  |  |  |  |
| TMH3 | Conserved Cys forms disulphide bridge to ECL2 | - | - | - | - | - |
|  | Second disulphide bridge to ECL2 | - |  |  |  |  |
|  | Gly bend |  |  |  | - | - |
| ICL2 | Helix - Indicated by Arg in TMH4 and Tyr in ICL2.* | + | + |  |  |  |
| TMH4 | Pro distortion | - | - | - | - | - |
|  | Bulge due to insertion |  | - | - |  |  |
| ECL2 | Disulphide bridge to TMH3 | - | - | - | - | - |
|  | Beta-sheet |  |  |  | - | - |
|  | Intra-ECL2 disulphide bridge |  | - | - |  |  |
|  | Alpha-helix – indicated by above disulphide bridge |  | - | - |  |  |
|  | Disulphide bridge to ECL1 | - |  |  |  |  |
|  | Beta-strand – indicated by above disulphide bridge | - |  |  |  |  |
|  | Second disulphide bridge to TMH3 | - |  |  |  |  |
| TMH5 | Pro distortion | - | - | - | - | - |
|  | Helix extension |  |  |  | ? |  |
| TMH6 | Pro distortion | + | + | + | + | + |
|  | Helix extension |  |  |  | ? |  |
| ECL3 | TMH6-ECL3 disulphide bridge** | + |  |  |  |  |
|  | 3_10_ helix – indicated by Glu sidechain forming H-bond to mainchain amide group (helix-capping). |  |  |  | - |  |
| TMH7 | Pro distortion | + | + | + | + | + |
| Helix 8 | Insertion | + |  |  | + | + |
|  |  |  |  |  |  |  |

+ indicates that hMC4R has a structural feature that is observed in a particular template(s).

- indicates that hMC4R does not have a structural feature that is observed in a particular template(s)

? indicates that there is uncertainty about whether hMC4R has a structural feature that is observed in a particular template(s).

* However sequence not similar

** The Cys residues are in different positions to Cystine in hAA2AR

**Table VIII:** **Structural features predicted to be present in hCNR1 based on sequence comparison to the five template GPCRs.**

|  |  | **hAA2AR** | **tB1AR** | **hB2AR** | **sRHO** | **bRHO** |
| --- | --- | --- | --- | --- | --- | --- |
| TMH1 | Pro distortion |  |  |  |  | - |
|  | Gly-Gly bulge |  |  |  | - |  |
| ICL1 | 3_10_ helix indicated by proton donor sidechain forming H-bond to acceptor group in helix 8. Followed by Leu. | - | - | - |  |  |
| TMH2 | Pro distortion | - | - | - | - |  |
|  | Gly-Gly distortion |  |  |  |  | - |
|  | Bulge due to insertion |  |  |  | + |  |
| ECL1 | Disulphide bridge to ECL2 | - |  |  |  |  |
|  | Beta-sheet structure (indicated by above disulphide bridge) | - |  |  |  |  |
| TMH3 | Conserved Cys forms disulphide bridge to ECL2 | - | - | - | - | - |
|  | Second disulphide bridge to ECL2 | - |  |  |  |  |
|  | Gly bend |  |  |  | - | - |
| ICL2 | Helix - Indicated by Arg in TMH4 and Tyr in ICL2. | - | - |  |  |  |
| TMH4 | Pro distortion | + | + | + | + | + |
|  | Bulge due to insertion |  | - | - |  |  |
| ECL2 | Disulphide bridge to TMH3 | - | - | - | - | - |
|  | Beta-sheet |  |  |  | ? | ? |
|  | Intra-ECL2 disulphide bridge* |  | + | + |  |  |
|  | Alpha-helix – indicated by above disulphide bridge |  | - | - |  |  |
|  | Disulphide bridge to ECL1 | - |  |  |  |  |
|  | Beta-strand – indicated by above disulphide bridge | - |  |  |  |  |
|  | Second disulphide bridge to TMH3 | - |  |  |  |  |
| TMH5 | Pro distortion | - | - | - | - | - |
|  | Helix extension |  |  |  | ? |  |
| TMH6 | Pro distortion | + | + | + | + | + |
|  | Helix extension |  |  |  | ? |  |
| ECL3 | TMH6-ECL3 disulphide bridge | - |  |  |  |  |
|  | 3_10_ helix – indicated by Glu sidechain forming H-bond to mainchain amide group (helix-capping). |  |  |  | - |  |
| TMH7 | Pro distortion | + | + | + | + | + |
| Helix 8 | Insertion | + |  |  | + | + |
|  |  |  |  |  |  |  |

+ indicates that hCNR1has a structural feature that is observed in a particular template(s).

- indicates that hCNR1does not have a structural feature that is observed in a particular template(s)

? indicates that there is uncertainty about whether hCNR1has a structural feature that is observed in a particular template(s).

* The Cys residues are in different positions to Cystine in tB1AR and hB2AR.

**Table IX:** **Structural features predicted to be present in hCNR2 based on sequence comparison to the five template GPCRs.**

|  |  | **hAA2AR** | **tB1AR** | **hB2AR** | **sRHO** | **bRHO** |
| --- | --- | --- | --- | --- | --- | --- |
| TMH1 | Pro distortion |  |  |  |  | - |
|  | Gly-Gly bulge |  |  |  | - |  |
| ICL1 | 3_10_ helix indicated by proton donor sidechain forming H-bond to acceptor group in helix 8. Followed by Leu. | - | - | - |  |  |
| TMH2 | Pro distortion | - | - | - | - |  |
|  | Gly-Gly distortion |  |  |  |  | - |
|  | Bulge due to insertion |  |  |  | + |  |
| ECL1 | Disulphide bridge to ECL2 | - |  |  |  |  |
|  | Beta-sheet structure (indicated by above disulphide bridge) | - |  |  |  |  |
| TMH3 | Conserved Cys forms disulphide bridge to ECL2 | - | - | - | - | - |
|  | Second disulphide bridge to ECL2 | - |  |  |  |  |
|  | Gly bend |  |  |  | - | - |
| ICL2 | Helix - Indicated by Arg in TMH4 and Tyr in ICL2.* | + | + |  |  |  |
| TMH4 | Pro distortion | + | + | + | + | + |
|  | Bulge due to insertion |  | - | - |  |  |
| ECL2 | Disulphide bridge to TMH3 | - | - | - | - | - |
|  | Beta-sheet |  |  |  | ? | ? |
|  | Intra-ECL2 disulphide bridge** |  | + | + |  |  |
|  | Alpha-helix – indicated by above disulphide bridge |  | - | - |  |  |
|  | Disulphide bridge to ECL1 | - |  |  |  |  |
|  | Beta-strand – indicated by above disulphide bridge | - |  |  |  |  |
|  | Second disulphide bridge to TMH3 | - |  |  |  |  |
| TMH5 | Pro distortion | - | - | - | - | - |
|  | Helix extension |  |  |  | ? |  |
| TMH6 | Pro distortion | + | + | + | + | + |
|  | Helix extension |  |  |  | ? |  |
| ECL3 | TMH6-ECL3 disulphide bridge | - |  |  |  |  |
|  | 3_10_ helix – indicated by Glu sidechain forming H-bond to mainchain amide group (helix-capping). |  |  |  | - |  |
| TMH7 | Pro distortion | + | + | + | + | + |
| Helix 8 | Insertion | + |  |  | + | + |
|  |  |  |  |  |  |  |

+ indicates that hCNR2 has a structural feature that is observed in a particular template(s).

- indicates that hCNR2 does not have a structural feature that is observed in a particular template(s)

? indicates that there is uncertainty about whether hCNR2 has a structural feature that is observed in a particular template(s).

* Sequences not similar

** The Cys residues are in different positions Cystine in tB1AR and hB2AR

**Table X: Structural features predicted to be present in hP2RY1 based on sequence comparison to the five template GPCRs.**

|  |  | **hAA2AR** | **tB1AR** | **hB2AR** | **sRHO** | **bRHO** |
| --- | --- | --- | --- | --- | --- | --- |
| TMH1 | Pro distortion |  |  |  |  | - |
|  | Gly-Gly bulge |  |  |  | - |  |
| ICL1 | 3_10_ helix indicated by proton donor sidechain forming H-bond to acceptor group in helix 8. Followed by Leu. | - | - | - |  |  |
| TMH2 | Pro distortion | + | + | + | + |  |
|  | Gly-Gly distortion |  |  |  |  | - |
|  | Bulge due to insertion |  |  |  | - |  |
| ECL1 | Disulphide bridge to ECL2 | - |  |  |  |  |
|  | Beta-sheet structure (indicated by above disulphide bridge) | - |  |  |  |  |
| TMH3 | Conserved Cys forms disulphide bridge to ECL2 | + | + | + | + | + |
|  | Second disulphide bridge to ECL2 | - |  |  |  |  |
|  | Gly bend |  |  |  | - | - |
| ICL2 | Helix - Indicated by Arg in TMH4 and Tyr in ICL2. | - | - |  |  |  |
| TMH4 | Pro distortion | + | + | + | + | + |
|  | Bulge due to insertion |  | - | - |  |  |
| ECL2 | Disulphide bridge to TMH3 | + | + | + | + | + |
|  | Beta-sheet |  |  |  | ? | ? |
|  | Intra-ECL2 disulphide bridge |  | - | - |  |  |
|  | Alpha-helix – indicated by above disulphide bridge |  | - | - |  |  |
|  | Disulphide bridge to ECL1 | - |  |  |  |  |
|  | Beta-strand – indicated by above disulphide bridge | - |  |  |  |  |
|  | Second disulphide bridge to TMH3 | - |  |  |  |  |
| TMH5 | Pro distortion | + | + | + | + | + |
|  | Helix extension |  |  |  | - |  |
| TMH6 | Pro distortion | + | + | + | + | + |
|  | Helix extension |  |  |  | - |  |
| ECL3 | TMH6-ECL3 disulphide bridge | - |  |  |  |  |
|  | 3_10_ helix – indicated by Glu sidechain forming H-bond to mainchain amide group (helix-capping). |  |  |  | ? |  |
| TMH7 | Pro distortion | + | + | + | + | + |
| Helix 8 | Insertion | + |  |  | + | + |
|  |  |  |  |  |  |  |

+ indicates that hP2RY1 has a structural feature that is observed in a particular template(s).

- indicates that hP2RY1 does not have a structural feature that is observed in a particular template(s)

? indicates that there is uncertainty about whether hP2RY1 has a structural feature that is observed in a particular template(s).

**Table XI:** **Structural features predicted to be present in hP2RY12 based on sequence comparison to the five template GPCRs.**

|  |  | **hAA2AR** | **tB1AR** | **hB2AR** | **sRHO** | **bRHO** |
| --- | --- | --- | --- | --- | --- | --- |
| TMH1 | Pro distortion |  |  |  |  | - |
|  | Gly-Gly bulge |  |  |  | - |  |
| ICL1 | 3_10_ helix indicated by proton donor sidechain forming H-bond to acceptor group in helix 8. Followed by Leu. | - | - | - |  |  |
| TMH2 | Pro distortion | + | + | + | + |  |
|  | Gly-Gly distortion |  |  |  |  | - |
|  | Bulge due to insertion |  |  |  | - |  |
| ECL1 | Disulphide bridge to ECL2 | - |  |  |  |  |
|  | Beta-sheet structure (indicated by above disulphide bridge) | - |  |  |  |  |
| TMH3 | Conserved Cys forms disulphide bridge to ECL2 | + | + | + | + | + |
|  | Second disulphide bridge to ECL2 | - |  |  |  |  |
|  | Gly bend |  |  |  | - | - |
| ICL2 | Helix - Indicated by Arg in TMH4 and Tyr in ICL2. | - | - |  |  |  |
| TMH4 | Pro distortion | + | + | + | + | + |
|  | Bulge due to insertion |  | - | - |  |  |
| ECL2 | Disulphide bridge to TMH3 | + | + | + | + | + |
|  | Beta-sheet |  |  |  | ? | ? |
|  | Intra-ECL2 disulphide bridge |  | - | - |  |  |
|  | Alpha-helix – indicated by Intra-ECL2 disulphide bridge |  | - | - |  |  |
|  | Disulphide bridge to ECL1 | - |  |  |  |  |
|  | Beta-strand – indicated by above disulphide bridge | - |  |  |  |  |
|  | Second disulphide bridge to TMH3 | - |  |  |  |  |
| TMH5 | Pro distortion | - | - | - | - | - |
|  | Helix extension |  |  |  | - |  |
| TMH6 | Pro distortion | + | + | + | + | + |
|  | Helix extension |  |  |  | - |  |
| ECL3 | TMH6-ECL3 disulphide bridge | - |  |  |  |  |
|  | 3_10_ helix – indicated by Glu sidechain forming H-bond to mainchain amide group (helix-capping). |  |  |  | ? |  |
| TMH7 | Pro distortion | + | + | + | + | + |
| Helix 8 | Insertion | + |  |  | + | + |
|  |  |  |  |  |  |  |

+ indicates that hP2RY12 has a structural feature that is observed in a particular template(s).

- indicates that hP2RY12 does not have a structural feature that is observed in a particular template(s)

? indicates that there is uncertainty about whether hP2RY12 has a structural feature that is observed in a particular template(s).

**Table XII:** **Structural features predicted to be present in hFSHR based on sequence comparison to the five template GPCRs.**

|  |  | **hAA2AR** | **tB1AR** | **hB2AR** | **sRHO** | **bRHO** |
| --- | --- | --- | --- | --- | --- | --- |
| TMH1 | Pro distortion |  |  |  |  | - |
|  | Gly-Gly bulge |  |  |  | - |  |
| ICL1 | 3_10_ helix indicated by proton donor sidechain forming H-bond to acceptor group in helix 8. Followed by Leu. | - | - | - |  |  |
| TMH2 | Pro distortion | - | - | - | - |  |
|  | Gly-Gly distortion |  |  |  |  | - |
|  | Bulge due to insertion |  |  |  | - |  |
| ECL1* | Disulphide bridge to ECL2 | - |  |  |  |  |
|  | Beta-sheet structure (indicated by above disulphide bridge) | - |  |  |  |  |
| TMH3 | Conserved Cys forms disulphide bridge to ECL2 | + | + | + | + | + |
|  | Second disulphide bridge to ECL2 | - |  |  |  |  |
|  | Gly bend |  |  |  | - | - |
| ICL2 | Helix - Indicated by Arg in TMH4 and Tyr in ICL2. | - | - |  |  |  |
| TMH4 | Pro distortion | + | + | + | + | + |
|  | Bulge due to insertion |  | - | - |  |  |
| ECL2 | Disulphide bridge to TMH3 | + | + | + | + | + |
|  | Beta-sheet |  |  |  | ? | ? |
|  | Intra-ECL2 disulphide bridge |  | - | - |  |  |
|  | Alpha-helix – indicated by Intra-ECL2 disulphide bridge |  | - | - |  |  |
|  | Disulphide bridge to ECL1 | - |  |  |  |  |
|  | Beta-strand – indicated by above disulphide bridge | - |  |  |  |  |
|  | Second disulphide bridge to TMH3 | - |  |  |  |  |
| TMH5 | Pro distortion | - | - | - | - | - |
|  | Helix extension |  |  |  | - |  |
| TMH6 | Pro distortion | + | + | + | + | + |
|  | Helix extension |  |  |  | - |  |
| ECL3 | TMH6-ECL3 disulphide bridge | - |  |  |  |  |
|  | 3_10_ helix – indicated by Glu sidechain forming H-bond to mainchain amide group (helix-capping). |  |  |  | - |  |
| TMH7 | Pro distortion | + | + | + | + | + |
| Helix 8 | Insertion | + |  |  | + | + |
|  |  |  |  |  |  |  |

+ indicates that hFSHR has a structural feature that is observed in a particular template(s).

- indicates that hFSHR does not have a structural feature that is observed in a particular template(s)

? indicates that there is uncertainty about whether hFSHR has a structural feature that is observed in a particular template(s).

* Very large compared to templates

**Table XIII:** **Structural features predicted to be present in hLHCGR based on sequence comparison to the five template GPCRs.**

|  |  | **hAA2AR** | **tB1AR** | **hB2AR** | **sRHO** | **bRHO** |
| --- | --- | --- | --- | --- | --- | --- |
| TMH1 | Pro distortion |  |  |  |  | - |
|  | Gly-Gly bulge |  |  |  | - |  |
| ICL1 | 3_10_ helix indicated by proton donor sidechain forming H-bond to acceptor group in helix 8. Followed by Leu. | - | - | - |  |  |
| TMH2 | Pro distortion | - | - | - | - |  |
|  | Gly-Gly distortion |  |  |  |  | - |
|  | Bulge due to insertion |  |  |  | - |  |
| ECL1* | Disulphide bridge to ECL2 | - |  |  |  |  |
|  | Beta-sheet structure (indicated by above disulphide bridge) | - |  |  |  |  |
| TMH3 | Conserved Cys forms disulphide bridge to ECL2 | + | + | + | + | + |
|  | Second disulphide bridge to ECL2 | - |  |  |  |  |
|  | Gly bend |  |  |  | - | - |
| ICL2 | Helix - Indicated by Arg in TMH4 and Tyr in ICL2. | - | - |  |  |  |
| TMH4 | Pro distortion | + | + | + | + | + |
|  | Bulge due to insertion |  | - | - |  |  |
| ECL2 | Disulphide bridge to TMH3 | + | + | + | + | + |
|  | Beta-sheet |  |  |  | ? | ? |
|  | Intra-ECL2 disulphide bridge |  | - | - |  |  |
|  | Alpha-helix – indicated by Intra-ECL2 disulphide bridge |  | - | - |  |  |
|  | Disulphide bridge to ECL1 | - |  |  |  |  |
|  | Beta-strand – indicated by above disulphide bridge | - |  |  |  |  |
|  | Second disulphide bridge to TMH3 | - |  |  |  |  |
| TMH5 | Pro distortion | - | - | - | - | - |
|  | Helix extension |  |  |  | - |  |
| TMH6 | Pro distortion | + | + | + | + | + |
|  | Helix extension |  |  |  | - |  |
| ECL3 | TMH6-ECL3 disulphide bridge | - |  |  |  |  |
|  | 3_10_ helix – indicated by Glu sidechain forming H-bond to mainchain amide group (helix-capping). |  |  |  | - |  |
| TMH7 | Pro distortion | + | + | + | + | + |
| Helix 8 | Insertion | + |  |  | + | + |
|  |  |  |  |  |  |  |

+ indicates that hLHCGR has a structural feature that is observed in a particular template(s).

- indicates that hLHCGR does not have a structural feature that is observed in a particular template(s)

? indicates that there is uncertainty about whether hLHCGR has a structural feature that is observed in a particular template(s).

* Very large compared to templates

**Table XIV:** **Structural features predicted to be present in rTSHR based on sequence comparison to the five template GPCRs.**

|  |  | **hAA2AR** | **tB1AR** | **hB2AR** | **sRHO** | **bRHO** |
| --- | --- | --- | --- | --- | --- | --- |
| TMH1 | Pro distortion |  |  |  |  | - |
|  | Gly-Gly bulge |  |  |  | - |  |
| ICL1 | 3_10_ helix indicated by proton donor sidechain forming H-bond to acceptor group in helix 8. Followed by Leu. | - | - | - |  |  |
| TMH2 | Pro distortion | - | - | - | - |  |
|  | Gly-Gly distortion |  |  |  |  | - |
|  | Bulge due to insertion |  |  |  | - |  |
| ECL1* | Disulphide bridge to ECL2 | - |  |  |  |  |
|  | Beta-sheet structure (indicated by above disulphide bridge) | - |  |  |  |  |
| TMH3 | Conserved Cys forms disulphide bridge to ECL2 | + | + | + | + | + |
|  | Second disulphide bridge to ECL2 | - |  |  |  |  |
|  | Gly bend |  |  |  | - | - |
| ICL2 | Helix - Indicated by Arg in TMH4 and Tyr in ICL2. | - | - |  |  |  |
| TMH4 | Pro distortion | + | + | + | + | + |
|  | Bulge due to insertion |  | - | - |  |  |
| ECL2 | Disulphide bridge to TMH3 | + | + | + | + | + |
|  | Beta-sheet |  |  |  | ? | ? |
|  | Intra-ECL2 disulphide bridge |  | - | - |  |  |
|  | Alpha-helix – indicated by Intra-ECL2 disulphide bridge |  | - | - |  |  |
|  | Disulphide bridge to ECL1 | - |  |  |  |  |
|  | Beta-strand – indicated by above disulphide bridge | - |  |  |  |  |
|  | Second disulphide bridge to TMH3 | - |  |  |  |  |
| TMH5 | Pro distortion | - | - | - | - | - |
|  | Helix extension |  |  |  | - |  |
| TMH6 | Pro distortion | + | + | + | + | + |
|  | Helix extension |  |  |  | - |  |
| ECL3 | TMH6-ECL3 disulphide bridge | - |  |  |  |  |
|  | 3_10_ helix – indicated by Glu sidechain forming H-bond to mainchain amide group (helix-capping). |  |  |  | - |  |
| TMH7 | Pro distortion | + | + | + | + | + |
| Helix 8 | Insertion | + |  |  | + | + |
|  |  |  |  |  |  |  |

+ indicates that hTSHR has a structural feature that is observed in a particular template(s).

- indicates that hTSHR does not have a structural feature that is observed in a particular template(s)

? indicates that there is uncertainty about whether hTSHR has a structural feature that is observed in a particular template(s).

* Very large compared to templates
