# Supplementary material for: Comparative Sequence and Structural Analyses of G-Protein-Coupled Receptor Crystal Structures and Implications for Molecular Models
Source: PLoS One. 2009 Sep 16;4(9):e7011. doi: 10.1371/journal.pone.0007011 (PMC2738427; doi:10.1371/journal.pone.0007011)
Supplement: Text S3 — The root mean squared deviation between each of the five template GPCR structures for TMH1, TMH3-7 and helix 8. (0.08 MB DOC) [file pone.0007011.s012.doc]

Supporting Text S3

The root mean squared deviation (RMSD) between each of the five template GPCR structures was calculated for each TMH and helix 8. All of these results (except those for TMH2 which are displayed in the accompanying paper) are shown below in Tables I to VII.

Table I: The RMSD of residues in TMH1

|  | **hAA2AR** | **tB1AR** | **hB2AR** | **sRHO** | **bRHO** |
| --- | --- | --- | --- | --- | --- |
| **hAA2AR** | 0.00 | 0.95 | 0.81 | 2.38 | 1.09 |
| **tB1AR** | 0.95 | 0.00 | 0.47 | 2.53 | 1.62 |
| **hB2AR** | 0.81 | 0.47 | 0.00 | 2.54 | 1.38 |
| **sRHO** | 2.38 | 2.53 | 2.54 | 0.00 | 2.34 |
| **bRHO** | 1.09 | 1.62 | 1.38 | 2.34 | 0.00 |

**Table II: The RMSD of residues in TMH3**

|  | **hAA2AR** | **tB1AR** | **hB2AR** | **sRHO** | **bRHO** |
| --- | --- | --- | --- | --- | --- |
| **hAA2AR** | 0.00 | 1.36 | 1.44 | 1.62 | 1.35 |
| **tB1AR** | 1.36 | 0.00 | 0.33 | 1.06 | 0.99 |
| **hB2AR** | 1.44 | 0.33 | 0.00 | 1.00 | 0.93 |
| **sRHO** | 1.62 | 1.06 | 1.00 | 0.00 | 0.88 |
| **bRHO** | 1.35 | 0.99 | 0.93 | 0.88 | 0.00 |

**Table III: The RMSD of residues in TMH4**

|  | **hAA2AR** | **tB1AR** | **hB2AR** | **sRHO** | **bRHO** |
| --- | --- | --- | --- | --- | --- |
| **hAA2AR** | 0.00 | 1.46 | 1.29 | 1.05 | 1.42 |
| **tB1AR** | 1.46 | 0.00 | 0.44 | 1.19 | 0.98 |
| **hB2AR** | 1.29 | 0.44 | 0.00 | 1.05 | 0.96 |
| **sRHO** | 1.05 | 1.19 | 1.05 | 0.00 | 0.79 |
| **bRHO** | 1.42 | 0.98 | 0.96 | 0.79 | 0.00 |

**Table IV: The RMSD of residues in TMH5**

|  | **hAA2AR** | **tB1AR** | **hB2AR** | **sRHO** | **bRHO** |
| --- | --- | --- | --- | --- | --- |
| **hAA2AR** | 0.00 | 1.53 | 1.49 | 1.57 | 1.52 |
| **tB1AR** | 1.53 | 0.00 | 0.26 | 0.84 | 0.84 |
| **hB2AR** | 1.49 | 0.26 | 0.00 | 0.85 | 0.90 |
| **sRHO** | 1.57 | 0.84 | 0.85 | 0.00 | 0.79 |
| **bRHO** | 1.52 | 0.84 | 0.90 | 0.79 | 0.00 |

**Table V: The RMSD of residues in TMH6**

|  | **hAA2AR** | **tB1AR** | **hB2AR** | **sRHO** | **bRHO** |
| --- | --- | --- | --- | --- | --- |
| **hAA2AR** | 0.00 | 1.17 | 1.07 | 1.11 | 1.34 |
| **tB1AR** | 1.17 | 0.00 | 0.44 | 0.93 | 0.99 |
| **hB2AR** | 1.07 | 0.44 | 0.00 | 0.78 | 0.96 |
| **sRHO** | 1.11 | 0.93 | 0.78 | 0.00 | 0.71 |
| **bRHO** | 1.34 | 0.99 | 0.96 | 0.71 | 0.00 |

**Table VI: The RMSD of residues in TMH7**

|  | **hAA2AR** | **tB1AR** | **hB2AR** | **sRHO** | **bRHO** |
| --- | --- | --- | --- | --- | --- |
| **hAA2AR** | 0.00 | 0.64 | 0.72 | 0.70 | 1.01 |
| **tB1AR** | 0.64 | 0.00 | 0.35 | 0.93 | 1.14 |
| **hB2AR** | 0.72 | 0.35 | 0.00 | 0.92 | 1.11 |
| **sRHO** | 0.70 | 0.93 | 0.92 | 0.00 | 0.83 |
| **bRHO** | 1.01 | 1.14 | 1.11 | 0.83 | 0.00 |

**Table VII: The RMSD of residues in Helix 8**

|  | **hAA2AR** | **tB1AR** | **hB2AR** | **sRHO** | **bRHO** |
| --- | --- | --- | --- | --- | --- |
| **hAA2AR** | 0.00 | 0.62 | 0.58 | 0.39 | 0.38 |
| **tB1AR** | 0.62 | 0.00 | 0.39 | 0.70 | 0.69 |
| **hB2AR** | 0.58 | 0.39 | 0.00 | 0.62 | 0.63 |
| **sRHO** | 0.39 | 0.70 | 0.62 | 0.00 | 0.48 |
| **bRHO** | 0.38 | 0.69 | 0.63 | 0.48 | 0.00 |
